# The Impact of Public Health and Social Measures (PHSMs) on SARS‐CoV‐2 Transmission in the WHO European Region (2020–2022)

**DOI:** 10.1111/irv.70036

**Published:** 2024-12-26

**Authors:** Yang Liu, Charlie Diamond, Sam Abbott, Kerry Wong, Tanja Schmidt, W. John Edmunds, Richard Pebody, Mark Jit

**Affiliations:** ^1^ Department of Infectious Disease Epidemiology, Faculty of Epidemiology and Population Health London School of Hygiene and Tropical Medicine London UK; ^2^ Centre for Mathematical Modelling of Infectious Diseases London School of Hygiene and Tropical Medicine London UK; ^3^ World Health Organization (WHO) Regional Office for Europe Copenhagen Denmark

**Keywords:** COVID‐19, evaluation, health policies, pandemic preparedness, preventative measures, public health and social measures, public health intervention, SARS‐CoV‐2

## Abstract

**Background:**

Between 2020 and 2022, countries used a range of different public health and social measures (PHSMs) to reduce the transmission of SARS‐CoV‐2. The impact of these PHSMs varied as the pandemic progressed, variants of concern (VOCs) emerged, vaccines rolled out and acceptance/uptake rates evolved. In this study, we assessed the impact of PHSMs in the World Health Organization (WHO) European Region during VOC phases.

**Methods:**

We relied on time series data on genome sequencing, PHSMs, health outcomes and physical contacts. Panel regression models were used to assess the association between PHSMs and SARS‐CoV‐2 transmission (approximated using time‐varying reproduction numbers). The interpretation of these regression models was assisted by hierarchical clustering, which was used to detect the temporal co‐occurrence of PHSMs. Generalised linear models were used to check if PHSMs are associated with physical contacts.

**Results:**

We identified four phases based on the dominating VOC in the WHO European Region: wild type (before early 2021), Alpha (early to mid‐2021), Delta (mid‐to‐late 2021) and Omicron (after late 2021). ‘School closure’, ‘stay‐at‐home requirement’ and ‘testing policy’ were consistently associated with lower transmission across VOC phases. The impact of most PHSMs varied by VOC phases without clear increasing or decreasing trends as the pandemic progressed. Several PHSMs associated with lower transmission were not associated with fewer physical contacts.

**Conclusions:**

The impact of PHSMs evolved as the pandemic progressed—although without clear trends. The specific mechanisms by which some PHSMs reduce SARS‐CoV‐2 transmission require further research.

## 1 Introduction

The ongoing COVID‐19 pandemic has had a large health burden around the world [1]. Most countries have implemented a wide range of public health and social measures (PHSMs) in response, defined by the World Health Organization (WHO) as ‘nonpharmaceutical interventions implemented by individuals, communities, and governments during health emergencies to reduce the risk and scale of transmission of infectious diseases [2]’, such as COVID‐19.

These PHSMs are generally designed to directly or indirectly reduce the frequency of physical contacts on which SARS‐CoV‐2 relies to transmit. PHSMs may have negative consequences on economic growth, mental well‐being and individual autonomy. Hence, understanding the impacts of different PHSMs is crucial both to retrospectively assess our response to the pandemic and to prepare for future pandemics.

Studies have used statistical models to look for temporal associations between PHSM intensity and markers of SARS‐CoV‐2 spread. However, this evidence has primarily focused on the impact of PHSMs over the initial epidemic waves, roughly spanning over the first three quarters of 2020 [3, 4, 5, 6]. There are several reasons why these estimates of PHSM impacts may not be extrapolated after late 2020. First, several variants of concerns (VOCs) emerged as the dominating strains regionally or globally. These strains may be more transmissible and may respond differently to existing immunity (either vaccine‐ or infection‐induced) compared to the strains which were dominant in early 2020. The changes in transmissibility may alter the implementation and impact of PHSMs drastically. Second, several COVID‐19 vaccines were approved beginning in late 2020 and subsequently achieved high coverage in some parts of the world [7]. Vaccination may change the impact of PHSMs because SARS‐CoV‐2 is not able to transmit as efficiently in a highly vaccinated population. High vaccine coverage may also allow a reduction in the stringency of PHSMs implemented by governments. Finally, the acceptance and uptake of PHSMs may change over time, driven by a wide range of factors, including familiarity with certain PHSMs, the availability of social security systems, the capacity of healthcare systems, risk perceptions and trust in authorities.

The WHO announced the end of COVID‐19 as a public health emergency of international concern in May 2023. There is a large volume of data on PHSM intensity systematically tracked across the world between 2020 and 2022, which provides us with an opportunity to retrospectively appraise the impact of PHSMs to inform future pandemic preparedness and outbreak response planning for both COVID‐19 and other pathogens. Here, we provide such appraisal specific to VOC phases for the WHO European Region, where data on PHSMs and COVID‐19 cases are relatively complete and where PHSM definitions are relatively comparable. We hypothesised that while PHSMs are effective in reducing SARS‐CoV‐2 transmission, the effectiveness changed as VOCs emerged and PHSM uptake varied. We further hypothesised that the effect mechanisms of these PHSMs involve reducing the number of physical contacts.

## 2 Methods

In this study, we used panel regression models to estimate the impact of PHSMs in reducing transmission of SARS‐CoV‐2. We divided the pandemic into four distinct phases according to the dominant VOC (i.e., wild type, Alpha, Delta and Omicron). We also used generalised linear models to explore the association between PHSMs and physical contacts since physical contacts are believed to be a mediator along the causal pathway that PHSMs take to reduce SARS‐CoV‐2 transmission. The interpretation of both regression models was assisted by temporal hierarchical clustering, which was used to identify the temporal co‐occurrence of PHSMs.

### 2.1 Data Sources

There are 53 Member States in the WHO European Region^1^—we could not include all Member States for a range of reasons that we will elaborate on in this section. We first estimated the time‐varying reproduction number (Rt) in each country to characterise the intensity of SARS‐CoV‐2 transmission using daily reported case counts extracted from *Our World in Data* [1]. This metric measures the number of secondary cases infected by an individual index case. We used a well‐established approach to estimate Rt [8], which has been described in detail elsewhere [9] and been used to assess the intensity of SARS‐CoV‐2 transmission [3, 10, 11]. In brief, this approach relied on deconvolution and known delay distributions (e.g., incubation period and onset‐to‐report delay) to reference cases back to their probable date of infection. The temporal changes in inferred daily infections (as opposed to observed reported cases) reflect the magnitude of Rt. The epidemiologic and healthcare system parameters we used (e.g., reporting delay, incubation period and generation time) were from the literature [12, 13, 14]. The Rt values used in this study have been visualised in Figures S1–S6.

The starting and ending dates of each VOC phase were determined using genome sequencing data from the Global Initiative on Sharing All Influenza Data (GISAID), accessed through the European Centre for Disease Prevention and Control [15]. VOC data have been collected weekly in this database. For the baseline analysis, we assume that a VOC phase starts if that VOC accounts for more than 30% of the samples tested that week. We varied this threshold from 10% to 50% with 10% increments as sensitivity analyses. We only kept country‐week combinations with more than 100 samples to mitigate the chance of random errors. With this criterion, the remaining dataset involved 30 of 53 countries in the WHO European Region. The same VOC phase definitions were used for the entire region (i.e., not country‐specific).

We downloaded the data on PHSMs from the Oxford COVID‐19 Government Response Tracker (OxCGRT, v.12). We focused only on the PHSMs that we believe to have direct short‐term causal pathways to reducing the transmission of SARS‐CoV‐2. Thus, PHSMs such as ‘investment in vaccines’ and ‘international support’ were not included. Thirteen PHSMs belonging to three categories (i.e., ‘Closure & Containment’, ‘Economic Response’ and ‘Public Health & Health System Reports’) were included in the analysis (see Table 1). All PHSMs were originally measured using ordinal categorical variables in the OxCGRT database. We rescaled them linearly to continuous variables between 0 and 1 to allow for comparability in the context of this study.

**TABLE 1:** Taxonomy of public health and social measures used in this study.

PHSM broad category	PHSM name
Internal containment and closure	School closures
	Workplace closures
	Cancellation of public events
	Limits on gathering sizes
	Closure of public transport
	Stay‐at‐home requirement
	Restrictions on internal movement
	International travel controls
Economic policies	Income support
	Debt/contract relief
Health system policies	Public information campaign
	Testing policy
	Contact tracing
	Facial covering policy

We further adjusted for COVID‐19 vaccination in the panel regression analysis. COVID‐19 vaccination may change the impact of PHSMs by modifying the acceptance and uptake of PHSMs. National‐level coverage of at least one dose of COVID‐19 was obtained from *Our World in Data* [7]. In the context of this study, we defined vaccine coverage using ‘people vaccinated (with >= 1 dose of COVID‐19) per hundred’ as we assume that the acceptance and uptake will start shifting as soon as individuals get vaccinated. Data on Rt, PHSMs and COVID‐19 vaccination simultaneously exist for 47 of 53 countries in the WHO European Region (apart from Armenia, Belarus, Montenegro, North Macedonia, Tajikistan and Turkmenistan), which were used in the panel regression models in this study.

We used the average number of daily reported contacts for all age groups as reported in the CoMix survey. CoMix is a multicountry study using weekly online surveys to collect data on individuals' physical contact patterns between the beginning of the pandemic and the end of 2022 [16, 17]. It was active for at least some of the time in more than 20 countries. In the context of this study, we only used contact data from countries with more than 3 months of contact history (i.e., 12 weeks)—longer time series are necessary for our statistical analysis associating PHSMs with physical contacts. CoMix survey results in Belgium, Finland, Germany, Lithuania, Malta, Netherlands (Kingdom of the), Switzerland and the United Kingdom were included in this part of the analysis.

### 2.2 Statistical Analysis

We applied hierarchical clustering techniques to the time series data of PHSMs to assess to what extent they were implemented with similar timing and intensity. We are interested in such temporal clustering of PHSMs as the effects of PHSMs that were always implemented simultaneously would not be independently identifiable. We used Ward's method, which seeks to minimise variance between clusters, to define the clusters and the Euclidean distance between time series to define the temporal distance between PHSMs [18, 19]. The hierarchical clustering process produced dendrograms, which are tree‐shaped graphs indicating if certain PHSMs belong to the same clusters. The statistical significance of clusters was assessed using multiscale bootstrapping (*n* = 10,000) and approximately unbiased and bootstrap probability *p*‐value thresholds of < 0.05.

We were not interested in cases where PHSMs were only similar in terms of timing. Therefore, we pruned the dendrograms resulting from the hierarchical clustering process. Since the objective function using Ward's method is the sum of squared errors, the pruning threshold of the dendrograms needed to be context‐specific. Thus, in this study, we pruned the dendrograms of the wild type and Omicron phases at 50 and of the Alpha and Delta phases at 25, broadly aligning with the lengths of these time series (Table 2).

**TABLE 2:** Date ranges for the different variant of concern phases.

Variant of concern phase	Phase start (uncertainty range)	Phase end (uncertainty range)	Duration (days) (uncertainty range)
Wild type	2020‐01‐01	2021‐01‐31 (2021‐01‐03 to 2021‐02‐15)	404 (369–411)
Alpha	2021‐02‐01 (2021‐01‐04 to 2021‐02‐16)	2021‐06‐13 (2021‐06‐07 to 2021‐06‐28)	133 (133–154)
Delta	2021‐06‐14 (2021‐06‐08 to 2021‐06‐29)	2021‐12‐19 (2021‐12‐13 to 2021‐12‐27)	182 (182–189)
Omicron	2021‐12‐20 (2021‐12‐12 to 2021‐12‐28)	2022‐12‐31	376 (369–383)

*Note:* The date ranges correspond to the baseline scenario, in which a variant of concern phase was assumed to have started when that variant accounted for more than 30% of all samples tested. The uncertainty range was obtained by varying this threshold between 10% and 50%, with 10% increments. There was no uncertainty around the starting date of the wild type phase or the ending date of the Omicron phase as these are constrained by the time horizon set for this analysis. These data ranges were based on genomic sequencing data available in 30 of 53 countries in the WHO European Region. More specific data inclusion criteria can be found in Section 2 above.

Linear models for panel data were used to investigate the association between PHSMs and SARS‐CoV‐2 transmission (proxied approximated by median Rt) over distinct VOC phases [20]. The model structure can be expressed as follows:

$${\mathrm{Rt}}_{\mathrm{it}}=\alpha_{i}+\sum \beta X_{\mathrm{it}}+\varepsilon_{\mathrm{it}}$$

where ${\mathrm{Rt}}_{\mathrm{it}}$ is the Rt of country *i* at time *t*, $\alpha_{i}$ is a country‐specific intercept (assumed to remain constant over each phase), $\sum \beta X_{t}$ represents the PHSMs and their corresponding coefficients and $\varepsilon_{\mathrm{it}}$ is the error term.

The above analytical framework requires the assumption of a causal link between the implementation of PHSMs and SARS‐CoV‐2 transmission in order to inform policy about interventions. From a public health management perspective, PHSMs have been designed to target the transmission pathways. In the case of COVID‐19 specifically, PHSMs have been designed to decrease effective contacts (i.e., physical contacts that could lead to infection). In this study, we further investigated to what degree PHSMs could influence physical contacts and how these associations varied over different VOC phases. A generalised linear model assuming Gamma distribution (with an ‘inverse’ link) was used. We included the VOC phase as an independent variable—both on its own and with interaction with PHSMs. Additional independent variables included country‐specific indicators (for country‐specific intercepts), contact settings (e.g., school and workplace) and PHSMs (apart from their interaction with VOC phases).

## 3 Results

### 3.1 Overview of Changes in PHSMs by VOC Phases

Based on sequencing data for the WHO European Region, we defined the date ranges of VOC phases as presented in Table 2. A detection threshold of 30% was used to identify the beginning of each phase. Varying this detection threshold from 10% to 50% led to changes in VOC phase markers by up to 4 weeks. The durations of the Alpha and Delta phases were substantially shorter than those of the wild type and Omicron phases.

**FIGURE 1:**
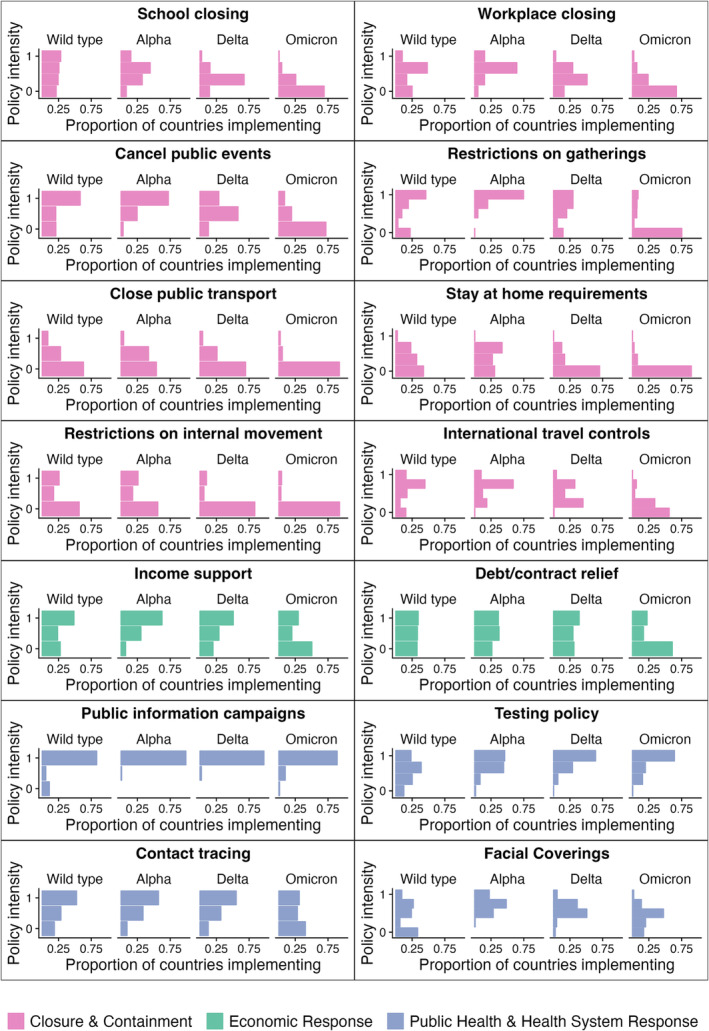
Prevalence of public health and social measures in the WHO European Region by variant of concern phases. Colours indicate general categories of public health and social measures. The numbers of intermediate levels between 0 and 1 differ by PHSM as they are rescaled from categorical variables with different levels between ‘not implemented’ and ‘implemented at the highest intensity possible.’

Highly disruptive PHSMs, such as the ‘closure of public transport’ and ‘restrictions on internal movements’, were among the least frequently used measures across VOC phases (Figure 1). The least disruptive PHSMs, such as ‘public health information campaigns’ and ‘testing policies’, came into place during the wild type or Alpha phases and have stayed at roughly the same level of intensity across VOC phases. Other PHSMs came into place during the wild type or Alpha phases and were gradually phased out during the Delta or Omicron phases.

**FIGURE 2:**
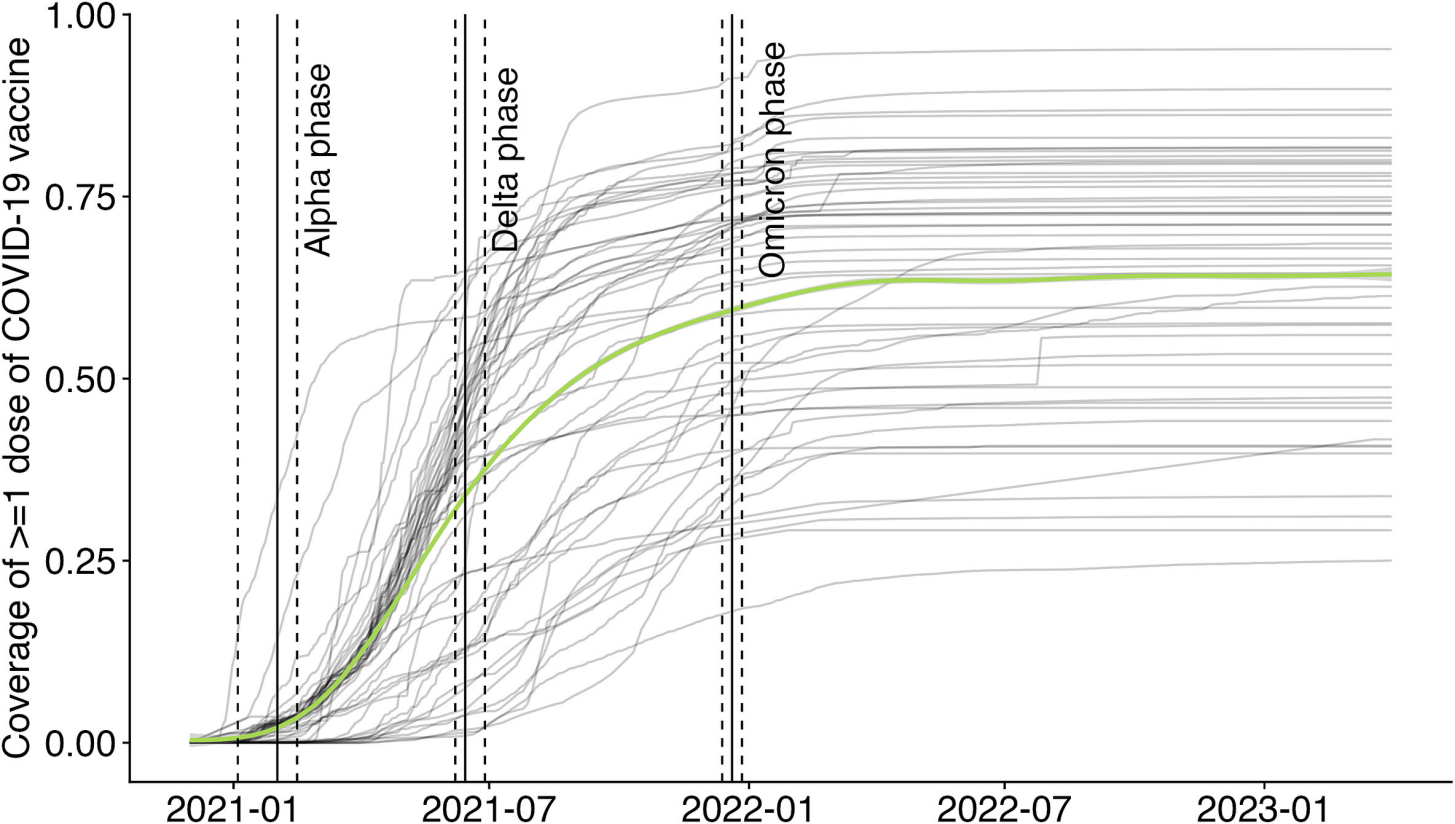
Coverage of > = 1 dose of COVID‐19 vaccine in the WHO European Region by country (grey lines) and across the entire region (green line). The solid vertical lines indicate the start of different variants of concern phases given the baseline scenario; the dashed lines indicate the uncertainty around the start/end of these phases. These uncertainty ranges are presented in Table 2.

The proportion of the population who have been vaccinated with at least one dose of COVID‐19 vaccines in the WHO European Region has substantially increased since the vaccines became available from the end of 2020 onwards (Figure 2). Most of such increases occurred during the Alpha and Delta phases. The fastest adopters (e.g., Israel and United Kingdom) received 60% coverage by the end of the Alpha phase. The coverage of at least one dose of COVID‐19 vaccines remained relatively low (<50%) for 13 countries a year into the Omicron phase.

### 3.2 Temporal Clustering Between PHSMs

**FIGURE 3:**
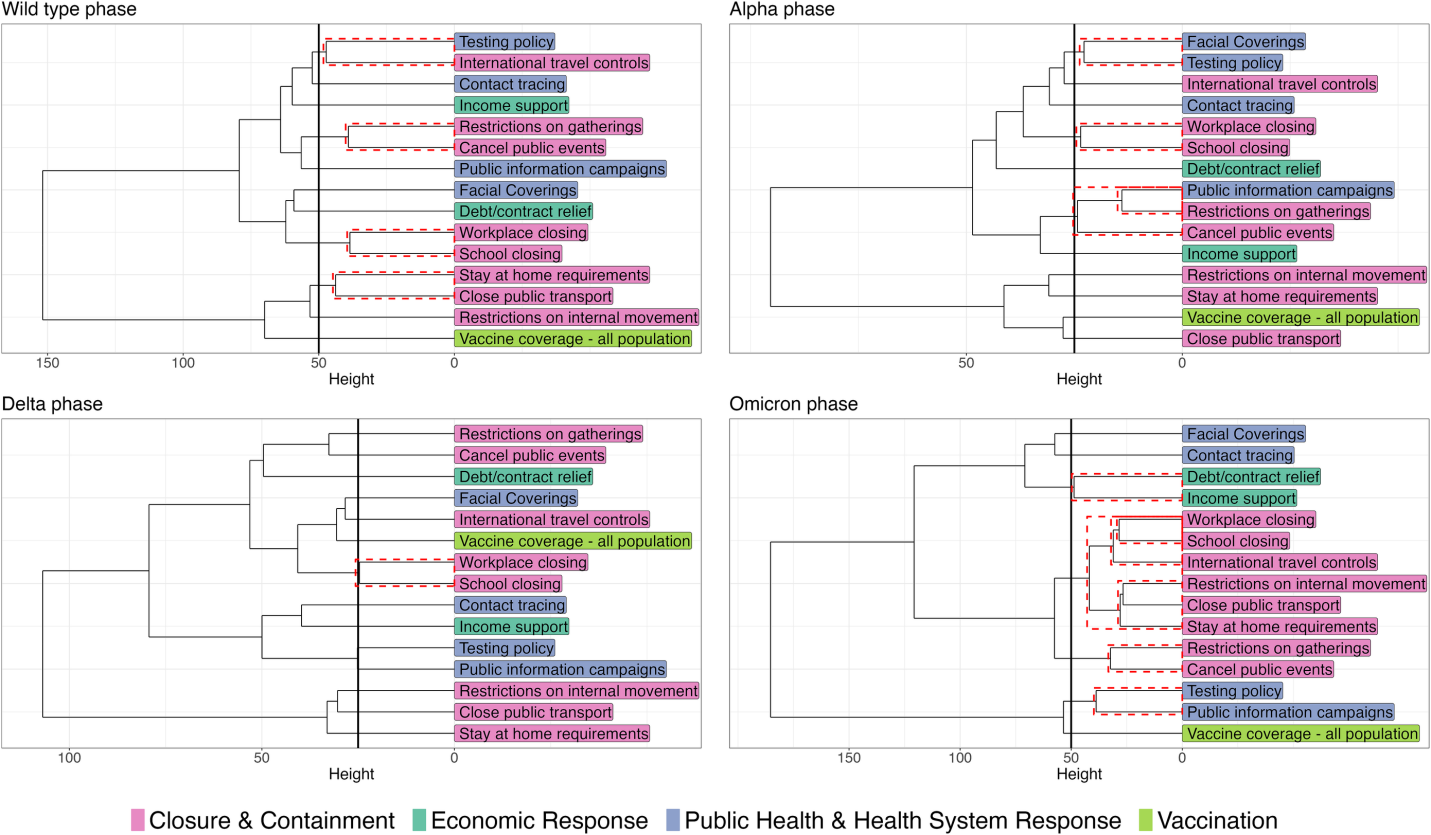
Temporal clusters by variant of concern phases. Dashed red boxes denote statistically significant temporal clusters based on bootstrapping.

The VOC phase with the highest degree of temporal clustering between PHSMs was the Omicron phase (Figure 3). During this phase, 12 of 15 PHSMs were members of a temporal cluster, and the largest temporal cluster contained six PHSMs, all of which belong to the ‘Closure & Containment’ category. This was a period of time when these PHSMs were slowly transitioned out in the WHO European Region (Figure 1). The VOC phase with the lowest degree of temporal clustering between PHSMs was the Delta phase. Only two PHSMs, ‘workplace closure’ and ‘school closure’ were in the same temporal cluster. In fact, these two PHSMs were in the same temporal cluster throughout the entire time series considered. The largest cluster size was two in both the Delta and the wild type phases—even if these PHSMs were in a temporal cluster, their effects would be relatively straightforward to interpret as only one other effect estimate would need to be taken into consideration. ‘Contact tracing’ and ‘vaccination’ were never in a temporal cluster with any other PHSMs. All observations made above were robust to changing thresholds that define VOC phases (Figures S7–S10).

### 3.3 Results From the Panel Regression Models

**FIGURE 4:**
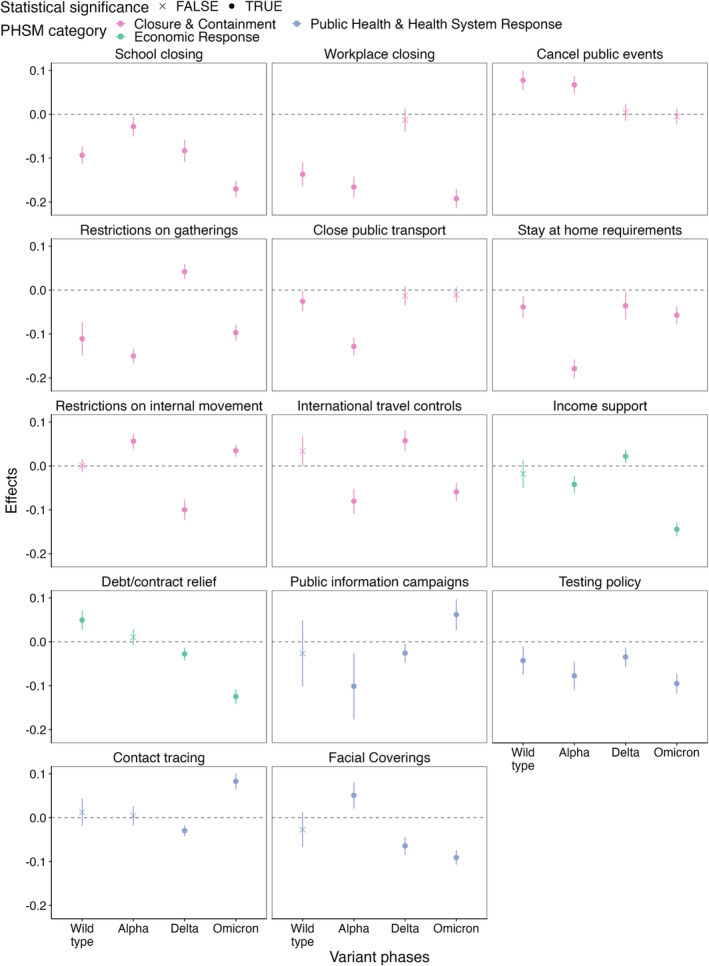
Effects of PHSMs on Rt by variants of concern phases.

The PHSMs with consistently significantly negative effect estimates on Rt were ‘school closure’, ‘stay‐at‐home requirement’ and ‘testing policy’ (Figure 4). The PHSMs with either negative or null effect estimates on Rt further included ‘workplace closure’ and ‘closure of public transport’. These effect estimates imply that the implementation of these PHSMs may be associated with lower Rt.

Some PHSMs have positive effect estimates. In other words, the implementation of these PHSMs was associated with higher Rt. Positive effect estimates observed in a temporal cluster may reflect residual confounding. Therefore, only positive estimates outside temporal clusters may indicate true positive effect estimates. PHSMs with positive effect estimates include ‘debt/contract relief’ during the wild type phase; ‘restrictions on internal movement’ during the Alpha phase; ‘restrictions on gatherings’, ‘international travel controls’ and ‘income support’ during the Delta phase; and ‘contact tracing’ during the Omicron phase.

While we observed substantial changes in terms of effect estimates over different VOC phases, we did not find substantial linear trends. In other words, the coefficients of most PHSMs did not consistently increase or decrease as the pandemic progressed. The only exceptions were ‘Debt/contract relief’ and ‘testing policy’—the coefficients of these PHSMs became more negative as the pandemic progressed. All observations made above were robust to changing thresholds that define VOC phases (Figures S11–S14).

### 3.4 The Impact of PHSMs on Physical Contacts

**FIGURE 5:**
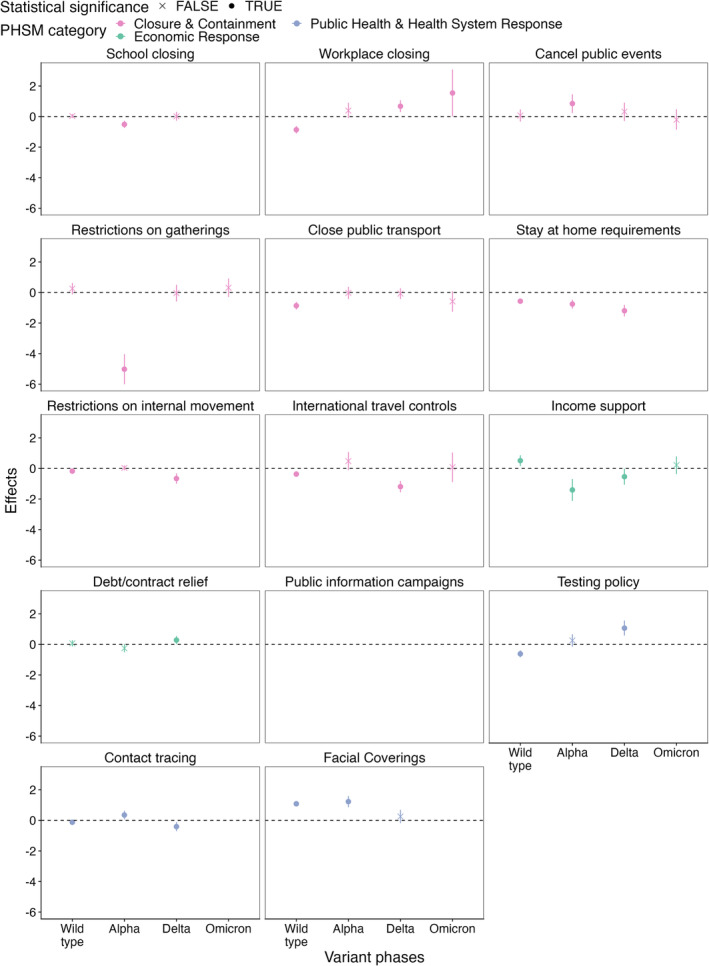
Effects of PHSMs on the number of physical contacts by variant of concern phases. We do not present effect estimates for ‘school closing’, ‘stay‐at‐home requirements’, ‘testing policy’ and ‘facial covering’ during the Omicron phase or the ‘public information campaign’ as there is enough variation in data to generate reliable inferences. For instance, the ‘public information campaign’ was active in all countries considered in this analysis during the entire study period; therefore, we do not have sufficient information to conclude the outcome when it is not active.

The only PHSM with consistently significantly negative effect estimates on physical contacts was the ‘stay‐at‐home requirement’ (Figure 5). The PHSMs with either negative or null effect estimates on physical contacts were ‘school closure’, ‘restriction on gatherings’, ‘closure of public transport’, ‘restrictions on internal movement’ and ‘international travel controls’. There was not sufficient variation in ‘public information campaigns’ to generate any reliable effect estimates on physical contacts throughout the pandemic. There was not sufficient variation in 7 of 15 PHSMs to generate any reliable effect estimates on physical contacts during the Omicron phase.

‘Face coverings’ and ‘income support’ were associated with higher numbers of physical contacts during the wild type phase. ‘Face covering’, ‘testing policy’ and ‘contact tracing’ were associated with higher numbers of physical contacts during the Alpha phase. ‘School closure’, ‘workplace closure’, ‘testing policy’ and ‘debt/contract relief’ were associated with higher numbers of physical contacts during the Delta phase.

Similar to the results from the panel regression models, we did not find consistent increases or diseasing among coefficients of most PHSMs as the pandemic progressed. The only exceptions were observed for ‘stay‐at‐home requirement’ and ‘testing policy’. As the pandemic progressed, ‘stay‐at‐home requirement’ was associated with lower frequencies in physical contacts, while ‘testing policy’ was associated with higher frequencies in physical contacts.

The direction of the association between PHSMs and Rt and the direction of the association between PHSMs and the frequency of physical contact were not necessarily the same. ‘Stay‐at‐home requirement’ was associated with fewer physical contacts and lower Rt. ‘Testing policy’, however, was associated with more physical contacts but lower Rt. For other PHSMs, the signals were complex. The cluster of ‘school closure’ and ‘workplace closure’ was associated with lower Rt as well as fewer physical contacts during the wild type and Alpha phases; during the Delta phase, it was associated with lower Rt but more physical contacts. Given that the results of ‘cancellation of public events’ and ‘facial covering’ need to be interpreted in their respective temporal clusters, we found no PHSM that was associated with both higher Rt and more physical contacts. ‘International travel controls’ were associated with higher Rt and fewer physical contacts. Compared to the association between PHSM and Rt, the association between PHSM and physical contacts by VOC phase is more sensitive to the detection threshold that defines when a VOC phase begins or ends (Figures S15–S18).

## 4 Discussion

In this study, we used hierarchical cluster analysis, panel regression models and generalised linear models to examine the association between PHSMs, the transmission of SARS‐CoV‐2 and physical contacts in the WHO European Region [21]. While single‐country analyses are able to factor in greater local contexts [22, 23], multicountry studies such as ours draw more data points from a greater variety of contexts, thus helping to augment the body of literature with generalisability, which is valuable to regional public health planning. The panel regression models we used (also referred to as longitudinal models) are one of several quantitative approaches that the research community has relied on to assess the impact of PHSMs [24, 25]. In this study, the interpretation of these models were further assisted by hierarchical cluster analysis, which helped us pick out potential structural confounding [3]. The results from the generalised linear models shed further light on the effect mechanism of PHSMs on Rt through physical contact changes, which addresses one of the key technical challenges of the field [26].

We looked at four distinct phases dominated by the wild type virus and the Alpha, Delta and Omicron variants to explore if the impact of PHSMs changed. Due to the changing VOCs and PHSM acceptance and uptake, we hypothesised that the impact of PHSMs would also have changed. The existing literature looking at the link between PHSMs and SARS‐CoV‐2 transmission in a multicountry context focuses on the pre‐Omicron phases, with the majority of the studies we found focusing on the first epidemic waves [4, 5].^2^ To our knowledge, our study is the first multicountry analysis to extend the study period to the Omicron phase (i.e., beyond the end of 2021).

There were significant temporal clustering between PHSMs in all VOC phases explored. Relative to other VOC phases, the Omicron phase had the largest number of PHSMs belonging to significant temporal clusters (12 of 15) and the largest number of PHSMs within a given temporal cluster (*n* = 6), making the isolation of effects for specific PHSMs challenging. This was a period when many PHSMs transitioned out. Consequently, the interpretation of the panel regression analysis during this phase requires the most caution. The Delta phase had the smallest number of PHSMs in significant temporal clusters (2 of 15). During the wild type and Delta phases, the largest temporal clusters only contained two members, making it less challenging to isolate the effects of individual PHSM.

Previous studies have suggested that workplace closures, restrictions on gatherings and cancellation of public events are among some of the most effective PHSMs at reducing the transmission of SARS‐CoV‐2 [27, 28, 29, 30, 31, 32]. Research with a greater focus on the European Region adds to this list ‘school closures’, travel restrictions and testing [33, 34, 35]. However, as we mentioned above, all of these studies have been based on pre‐Omicron or even wild type only phases. In this study, we found that ‘school closure’, ‘stay‐at‐home requirements’ and ‘testing policy’ were consistently associated with lower Rt throughout the entire pandemic phase we considered. ‘Workplace closure’ and ‘closure of public transport’ had either negative or null effect estimates on Rt. While there were changes in the effect estimates of PHSMs on predicting Rt, there was no consistently increasing or decreasing trend over time among the coefficients.

A small number of PHSMs, after factoring in temporal clustering (or the lack of), were associated with higher Rt during specific VOC phases. These results, while surprising, are not impossible. For example, ‘contact tracing’ is associated with higher case identification, which may appear as an increase in transmission; ‘restrictions on internal movement’ may modify local contact patterns as people spend more time around their residential area. These results need to be further validated using different datasets, and the underlying effect mechanisms need to be investigated.

Only the ‘stay‐at‐home requirement’ was consistently associated with fewer physical contacts. ‘School closure’, ‘restriction on gatherings’, ‘closure of public transport’, ‘restrictions on internal movements’ and ‘international travel controls’ were not associated or associated with fewer physical contacts. Similar to the models above, although there were changes in the effect estimates of PHSMs on predicting physical contacts, there was no clear increasing or decreasing trend over time among them. A small number of PHSMs, during specific VOC phases, were associated with higher numbers of physical contacts.

Most PHSMs were designed to reduce the transmission of SARS‐CoV‐2 in the community by reducing physical contact. Our results showed that only the ‘stay‐at‐home requirement’ consistently showed associations with both lower Rt and fewer physical contacts across all VOC phases. During the Delta phase, ‘testing policy’ and ‘Debt/contract relief’ were associated with lower Rt and more physical contacts. These PHSMs may have reduced the frequency of ‘effective contacts’ (i.e., contacts that resulted in transmission) and not general physical contacts. For ‘testing policy’ specifically, this may reflect individual decisions to socialise based on their test results [36]. These PHSMs may be able to reduce transmission without disrupting people's day‐to‐day lives, potentially making them ideal public health interventions in future pandemics.

‘School closure’ and ‘workplace closure’ consistently appeared in the same temporal cluster throughout the pandemic. During the initial phases (i.e., wild type and Alpha), they were associated with lower Rt and fewer physical contacts. During the Delta phase, however, they were associated with lower Rt but more physical contacts. The mechanisms by which these PHSMs influenced Rt may have changed over time. Surprisingly, ‘international travel controls’ were associated with higher Rt and fewer physical contacts during the Delta phase. There is a possibility of residual confounding—this PHSM was only used during the Delta phase when the potential introduction of a more transmissible VOC was high [37]. There may have also been unintended causal pathways that require further research to disentangle. However, no PHSM was associated with more physical contacts and higher Rt.

Our study has several limitations. First, we estimated the combined impact of PHSMs and their acceptance and uptake levels in this study as we do not have access to behaviour data that could help us separate them. Systematically collecting behaviour data may be crucial in future pandemic response efforts. Second, we used an observational study design. Most PHSMs we studied were in significant temporal clusters at some point during the COVID‐19 pandemic. In other words, they were implemented and lifted simultaneously with some other PHSMs, creating a situation where their effect sizes cannot be independently assessed. These factors reduce our ability to make accurate inferences and to establish causality. Hence, we limit our discussion to the direction of associations without discussing their magnitude or implying causality. Further, while discussing overall effectiveness, we only focus on PHSMs with consistent results in terms of the direction of associations. Third, we did not explore the effect of any interactions between PHSMs. For instance, workplace closure may be more effective when accompanied by income support. Fourth, the progression of epidemic waves within the WHO European Region has been relatively synchronised, the timing of variant phases has been similar and the PHSMs in use are also relatively similar. An expanded study that includes more countries may be useful in gaining a fuller picture. However, such an analysis may have other issues, as some PHSMs may not be comparable between regions. Last but not the least, there may have been changes in population representativeness in the CoMix study (used to approximate weekly changes in physical contact) that we could not account for in this study [16].

## 5 Conclusion

Among all PHSMs investigated in the current paper, ‘school closure’, ‘stay‐at‐home requirement’ and ‘testing policy’ were consistently associated with lower Rt. There was slightly weaker evidence that supports the impact of ‘workplace closure’ and ‘closure of public transport’ on SARS‐CoV‐2 transmission. These are PHSMs that may most likely be effective in future outbreak response if a pathogen with primary modes of transmission and epidemiological characteristics like SARS‐CoV‐2 emerges in the future, particularly before an effective vaccine is available or widely accessible.

The impact of the other PHSMs significantly varied by VOC phase, with no consistent increasing or decreasing trends in estimates as the pandemic progressed. Several PHSMs associated with lower Rt were not associated with fewer physical contacts. Their effect mechanisms need to be further investigated as part of future research and to inform optimal design of PHSMs, as we face future emerging epidemics.

## Author Contributions

**Yang Liu:** conceptualization, methodology, software, data curation, formal analysis, writing – original draft, writing – review and editing, visualization. **Charlie Diamond:** conceptualization, methodology, software, data curation, formal analysis, writing – original draft, writing – review and editing, visualization. **Sam Abbott:** methodology, data curation, formal analysis, writing – review and editing. **Kerry Wong:** data curation, writing – review and editing. **Tanja Schmidt:** writing – review and editing, validation. **W. John Edmunds:** data curation, writing – review and editing. **Richard Pebody:** conceptualization, writing–review and editing, visualization. **Mark Jit:** conceptualization, writing – review and editing, visualization.

## Conflicts of Interest

The authors declare no conflicts of interest.

### Peer Review

The peer review history for this article is available at https://www.webofscience.com/api/gateway/wos/peer‐review/10.1111/irv.70036.

## Supporting information


**Figure S1:** Rt results, panel 1. Countries starting from A to B.
**Figure S2:** Rt results, panel 2. Countries starting from C to G.
**Figure S3:** Rt results, panel 3. Countries starting from G to L.
**Figure S4:** Rt results, panel 4. Countries starting from L to N.
**Figure S5:** Rt results, panel 5. Countries starting from P to S.
**Figure S6:** Rt results, panel 6. Countries starting from S to U.
**Figure S7:** Temporal clusters by variant of concern phases. Dashed red boxes denote statistically significant temporal clusters based on bootstrapping. Detection threshold = 0.1.
**Figure S8:** Temporal clusters by variant of concern phases. Dashed red boxes denote statistically significant temporal clusters based on bootstrapping. Detection threshold = 0.2.
**Figure S9:** Temporal clusters by variant of concern phases. Dashed red boxes denote statistically significant temporal clusters based on bootstrapping. Detection threshold = 0.4.
**Figure S10:** Temporal clusters by variant of concern phases. Dashed red boxes denote statistically significant temporal clusters based on bootstrapping. Detection threshold = 0.5.
**Figure S11:** Effects by variants of concern phases for the association between PHSMs and Rt. Detection threshold = 0.1.
**Figure S12:** Effects by variants of concern phases for the association between PHSMs and Rt. Detection threshold = 0.2.
**Figure S13:** Effects by variants of concern phases for the association between PHSMs and Rt. Detection threshold = 0.4.
**Figure S14:** Effect by variants of concern phases for the association between PHSMs and Rt. Detection threshold = 0.5.
**Figure S15:** Effects by variants of concern phases for the association between PHSMs and physical contacts. Detection threshold = 0.1.
**Figure S16:** Effects by variants of concern phases for the association between PHSMs and physical contacts. Detection threshold = 0.2.
**Figure S17:** Effects by variants of concern phases for the association between PHSMs and physical contacts. Detection threshold = 0.4.
**Figure S18:** Effects by variants of concern phases for the association between PHSMs and physical contacts. Detection threshold = 0.5.

## Data Availability

This study relies solely on publicly available data. Complete references have been provided in the main text to facilitate access. All code used for this project can be downloaded via GitHub (https://github.com/yangclaraliu/NPI_WHO_EURO).

## Endnotes

1 Albania, Andorra, Armenia, Austria, Azerbaijan, Belarus, Belgium, Bosnia and Herzegovina, Bulgaria, Croatia, Cyprus, Czechia, Denmark, Estonia, Finland, France, Georgia, Germany, Greece, Hungary, Iceland, Ireland, Israel, Italy, Kazakhstan, Kyrgyzstan, Latvia, Lithuania, Luxembourg, Malta, Moldova, Monaco, Montenegro, Netherlands (Kingdom of the), North Macedonia, Norway, Poland, Portugal, Romania, Russia, San Marino, Serbia, Slovakia, Slovenia, Spain, Sweden, Switzerland, Tajikistan, Türkiye, Turkmenistan, Ukraine, United Kingdom, Uzbekistan.

^2^
Note that these PHSMs may be defined differently in these studies.
