# Supplementary material for: The Impact of Public Health and Social Measures (PHSMs) on SARS‐CoV‐2 Transmission in the WHO European Region (2020–2022)
Source: Influenza Other Respir Viruses. 2024 Dec 26;18(12):e70036. doi: 10.1111/irv.70036 (PMC11671160; doi:10.1111/irv.70036)

[Supplemental Material]

The impact of public health and social measures (PHSMs) on SARS-CoV-2 transmission in the WHO European Region (2020-2022)

[Figure S1: Rt results, panel 1](#_Toc178943096)

[Figure S2: Rt results, panel 2](#_Toc178943097)

[Figure S3: Rt results, panel 3](#_Toc178943098)

[Figure S4: Rt results, panel 4](#_Toc178943099)

[Figure S5: Rt results, panel 5](#_Toc178943100)

[Figure S6: Rt results, panel 6](#_Toc178943101)

[Figure S7: Temporal clusters by variant of concern phases. Dashed red boxes denote statistically significant temporal clusters based on bootstrapping. Detection threshold = 0.1.](#_Toc178943102)

[Figure S8: Temporal clusters by variant of concern phases. Dashed red boxes denote statistically significant temporal clusters based on bootstrapping. Detection threshold = 0.2.](#_Toc178943103)

[Figure S9: Temporal clusters by variant of concern phases. Dashed red boxes denote statistically significant temporal clusters based on bootstrapping. Detection threshold = 0.4.](#_Toc178943104)

[Figure S10: Temporal clusters by variant of concern phases. Dashed red boxes denote statistically significant temporal clusters based on bootstrapping. Detection threshold = 0.5.](#_Toc178943105)

[Figure S11: Effects by variants of concern phases for the association between PHSMs and Rt. Detection threshold = 0.1.](#_Toc178943106)

[Figure S12: Effects by variants of concern phases for the association between PHSMs and Rt. Detection threshold = 0.2.](#_Toc178943107)

[Figure S13: Effects by variants of concern phases for the association between PHSMs and Rt. Detection threshold = 0.4.](#_Toc178943108)

[Figure S14: Effect by variants of concern phases for the association between PHSMs and Rt. Detection threshold = 0.5.](#_Toc178943109)

[Figure S15: Effects by variants of concern phases for the association between PHSMs and physical contacts. Detection threshold = 0.1.](#_Toc178943110)

[Figure S16: Effects by variants of concern phases for the association between PHSMs and physical contacts. Detection threshold = 0.2.](#_Toc178943111)

[Figure S17: Effects by variants of concern phases for the association between PHSMs and physical contacts. Detection threshold = 0.4.](#_Toc178943112)

[Figure S18: Effects by variants of concern phases for the association between PHSMs and physical contacts. Detection threshold = 0.5.](#_Toc178943113)

### Figure S1: Rt results, panel 1


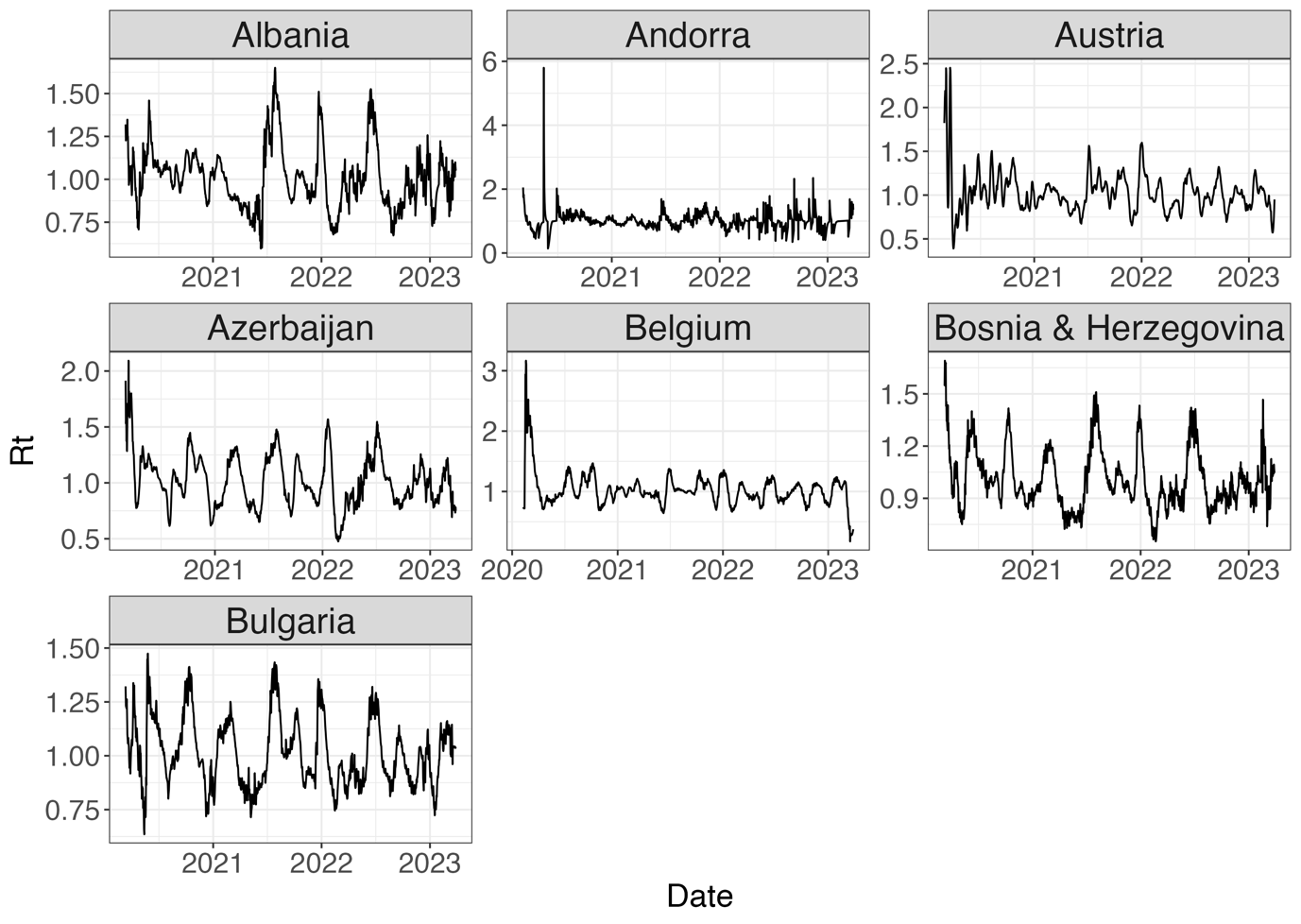


### Figure S2: Rt results, panel 2


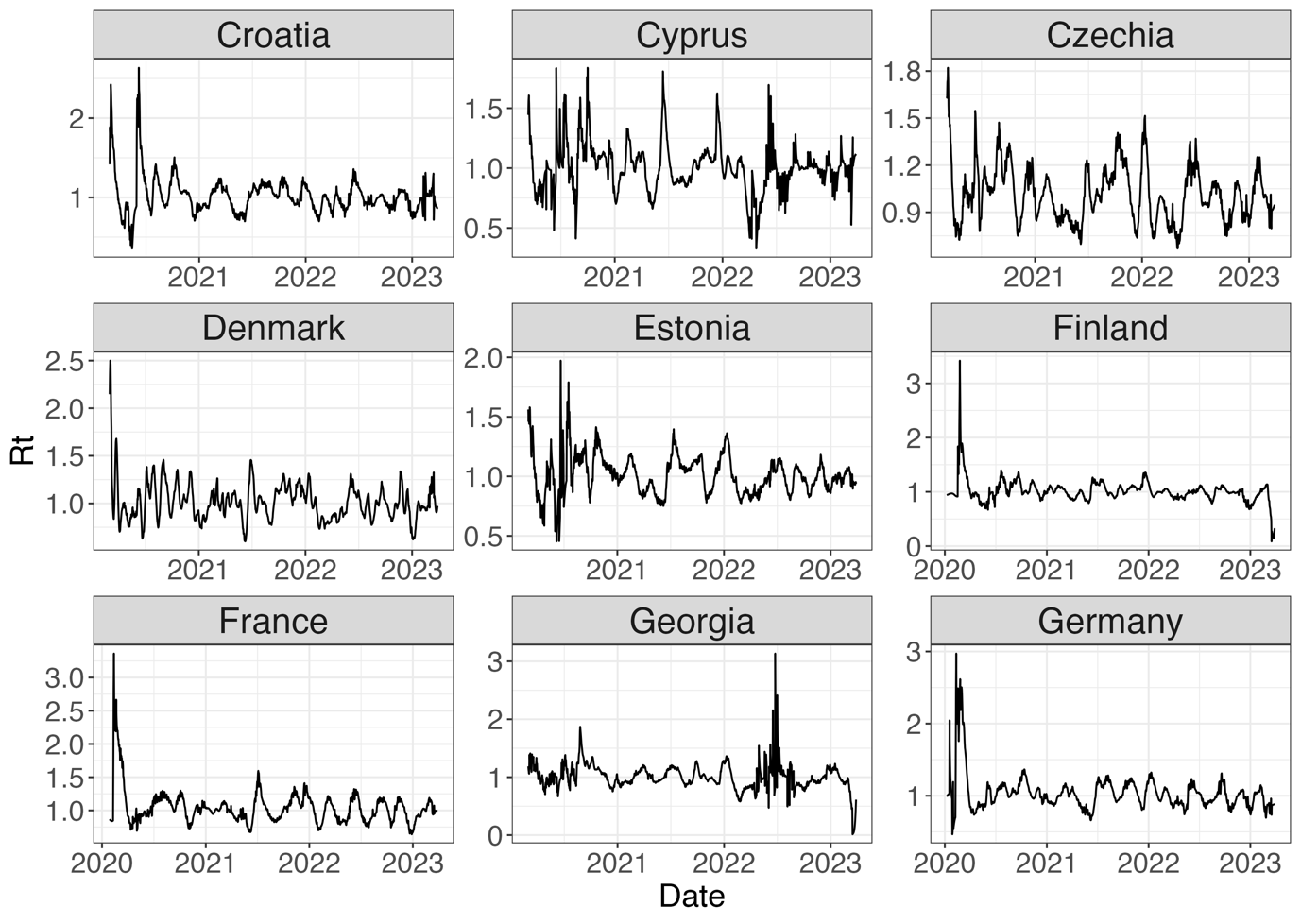


### Figure S3: Rt results, panel 3


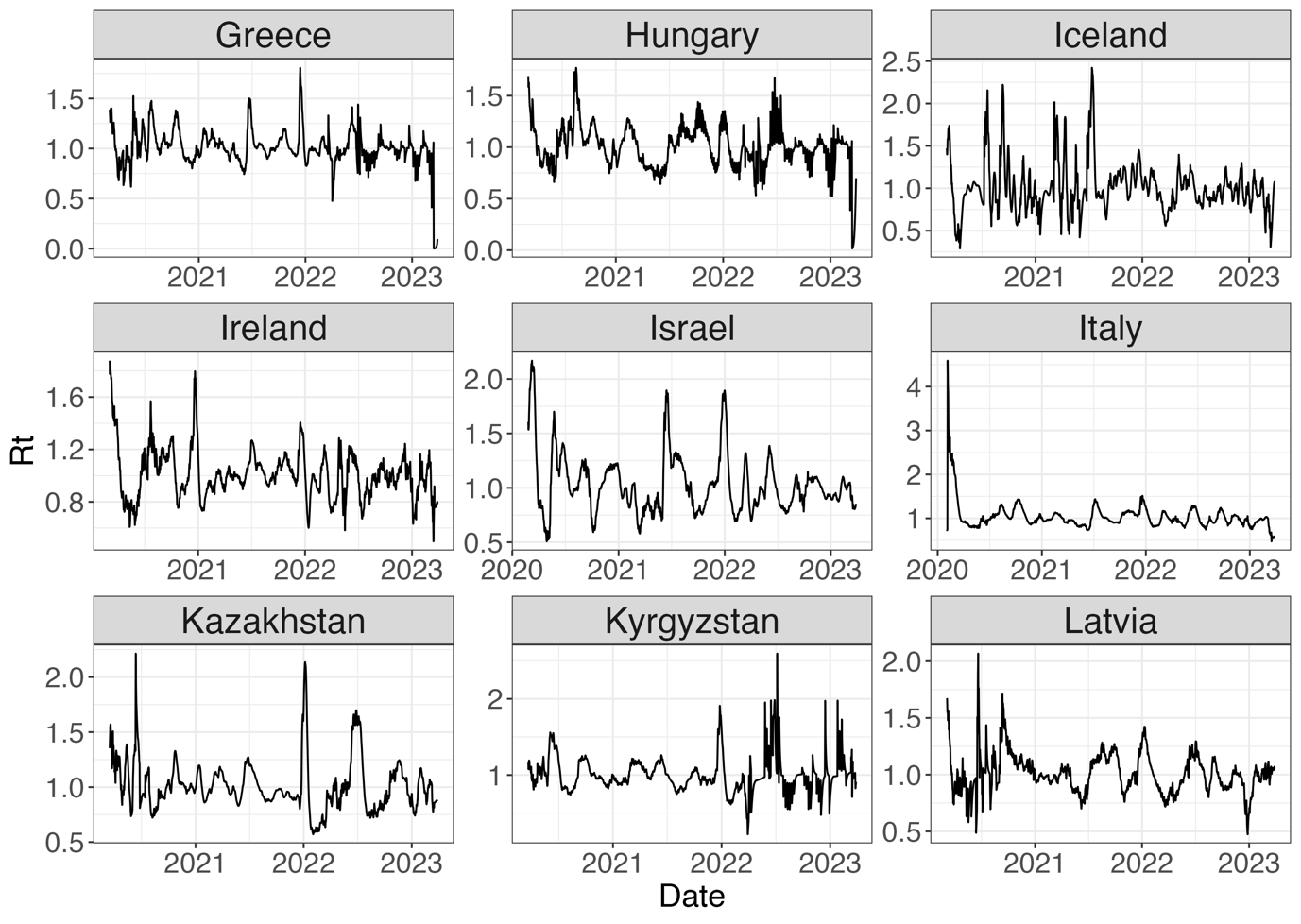


### Figure S4: Rt results, panel 4


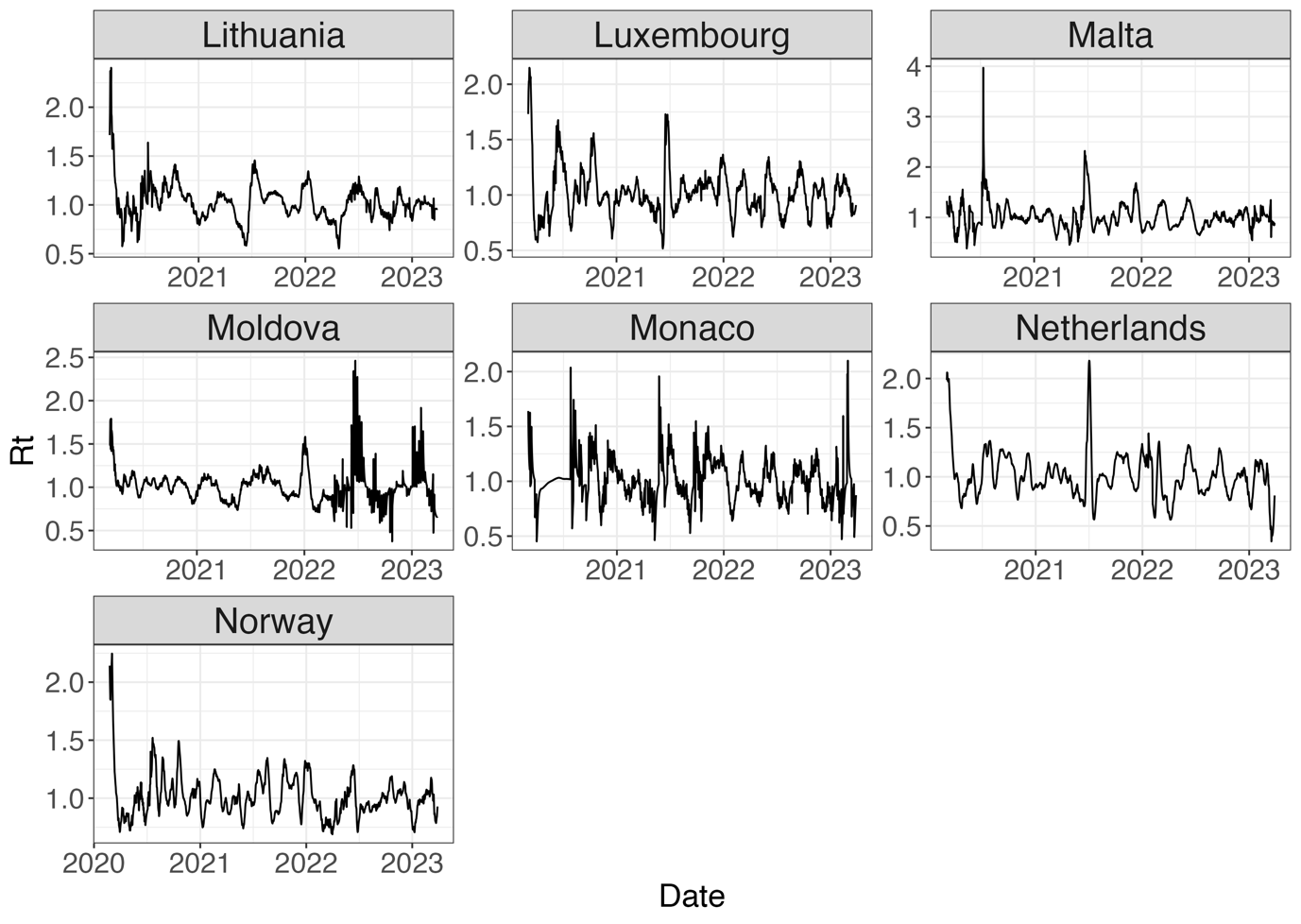


### Figure S5: Rt results, panel 5


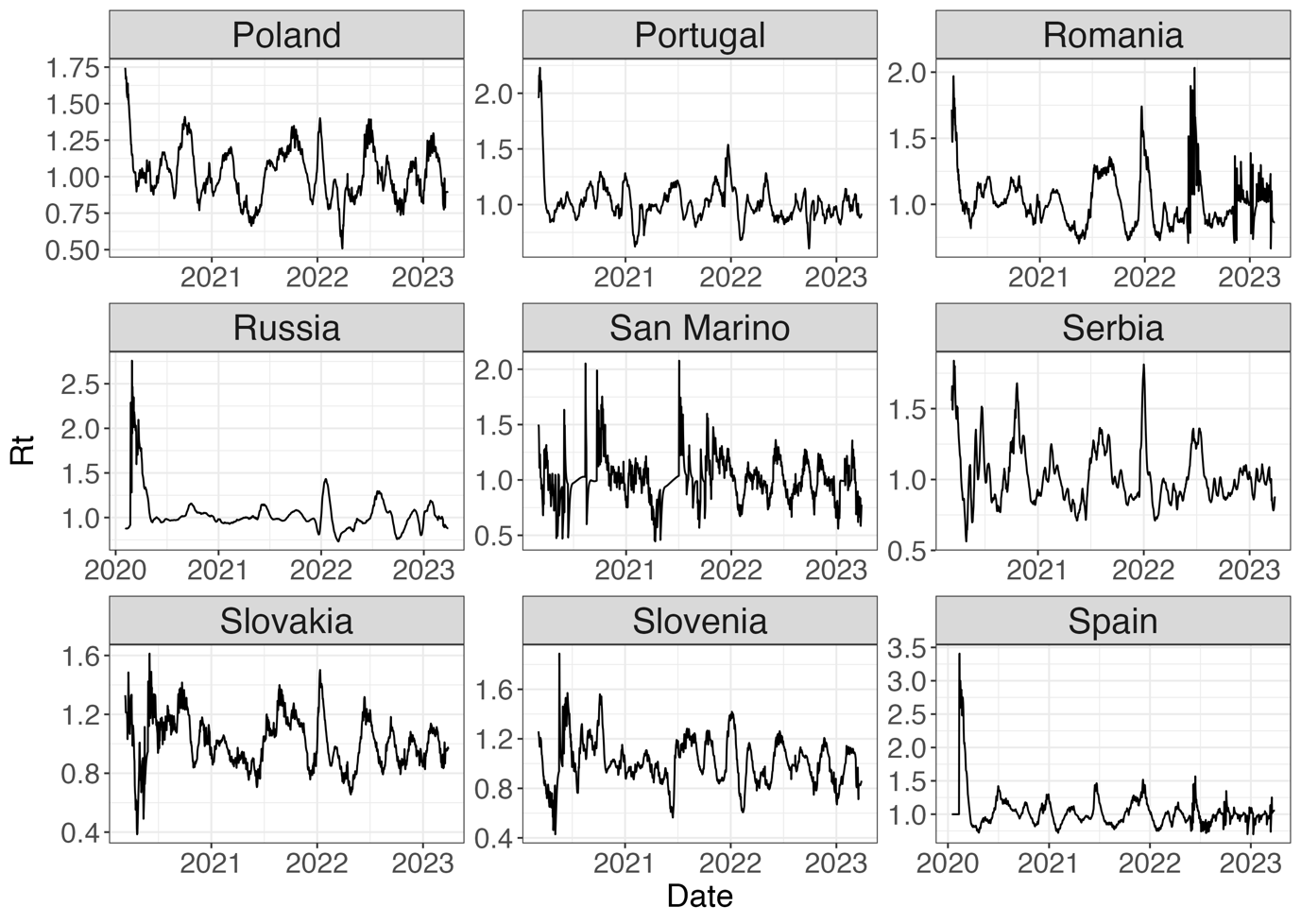


### Figure S6: Rt results, panel 6


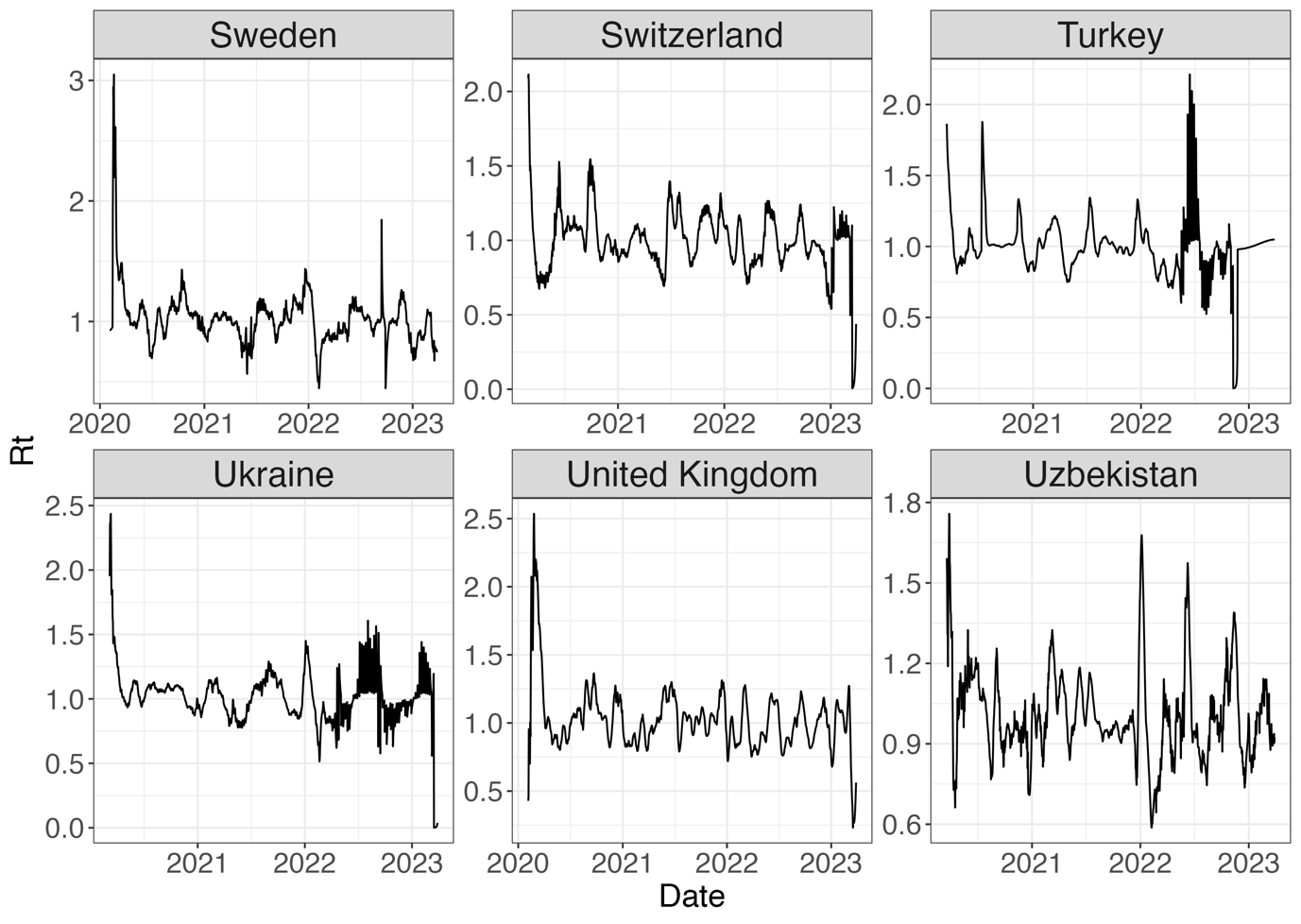


### Figure S7: Temporal clusters by variant of concern phases. Dashed red boxes denote statistically significant temporal clusters based on bootstrapping. Detection threshold = 0.1.


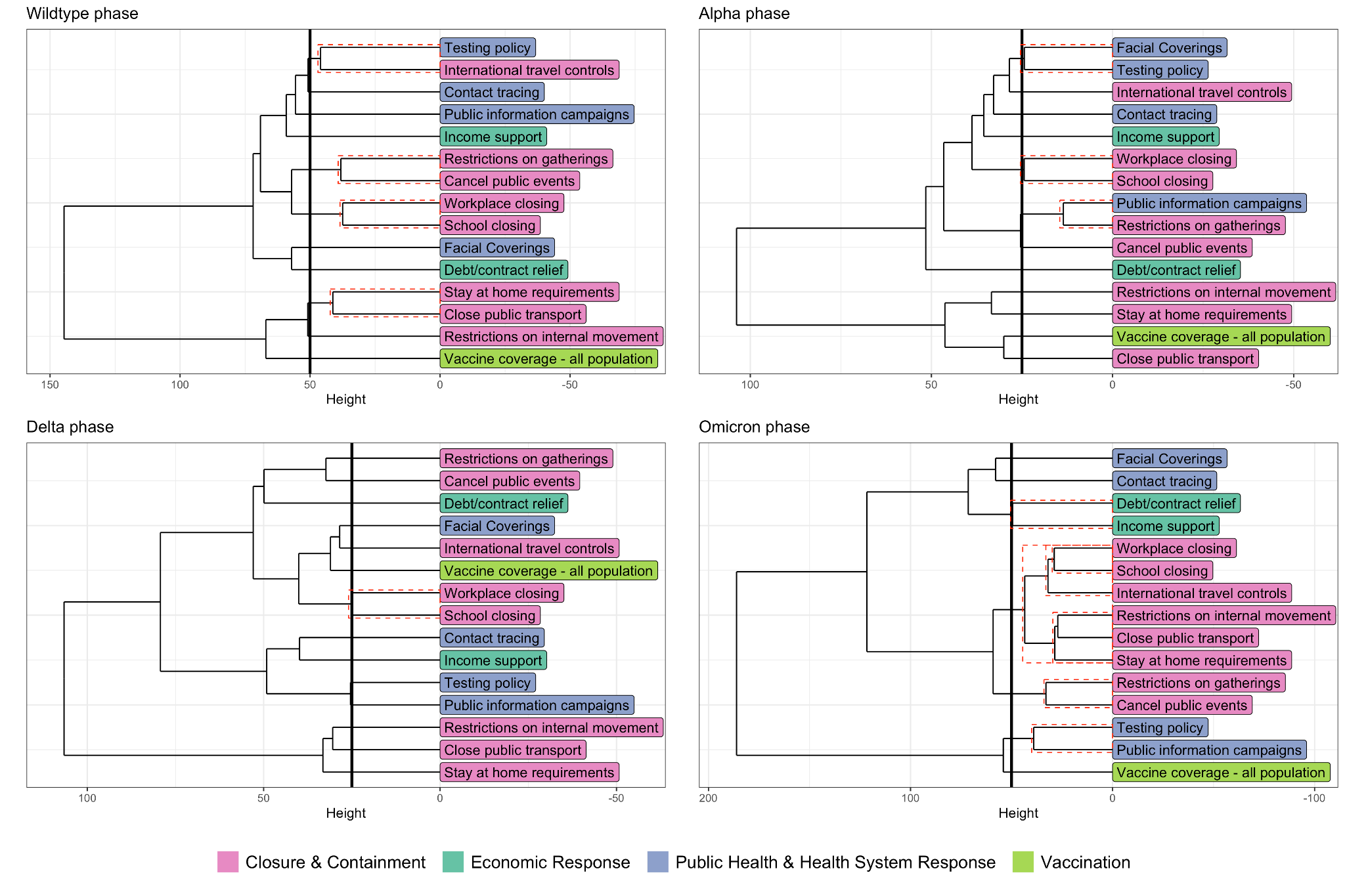


### Figure S8: Temporal clusters by variant of concern phases. Dashed red boxes denote statistically significant temporal clusters based on bootstrapping. Detection threshold = 0.2.


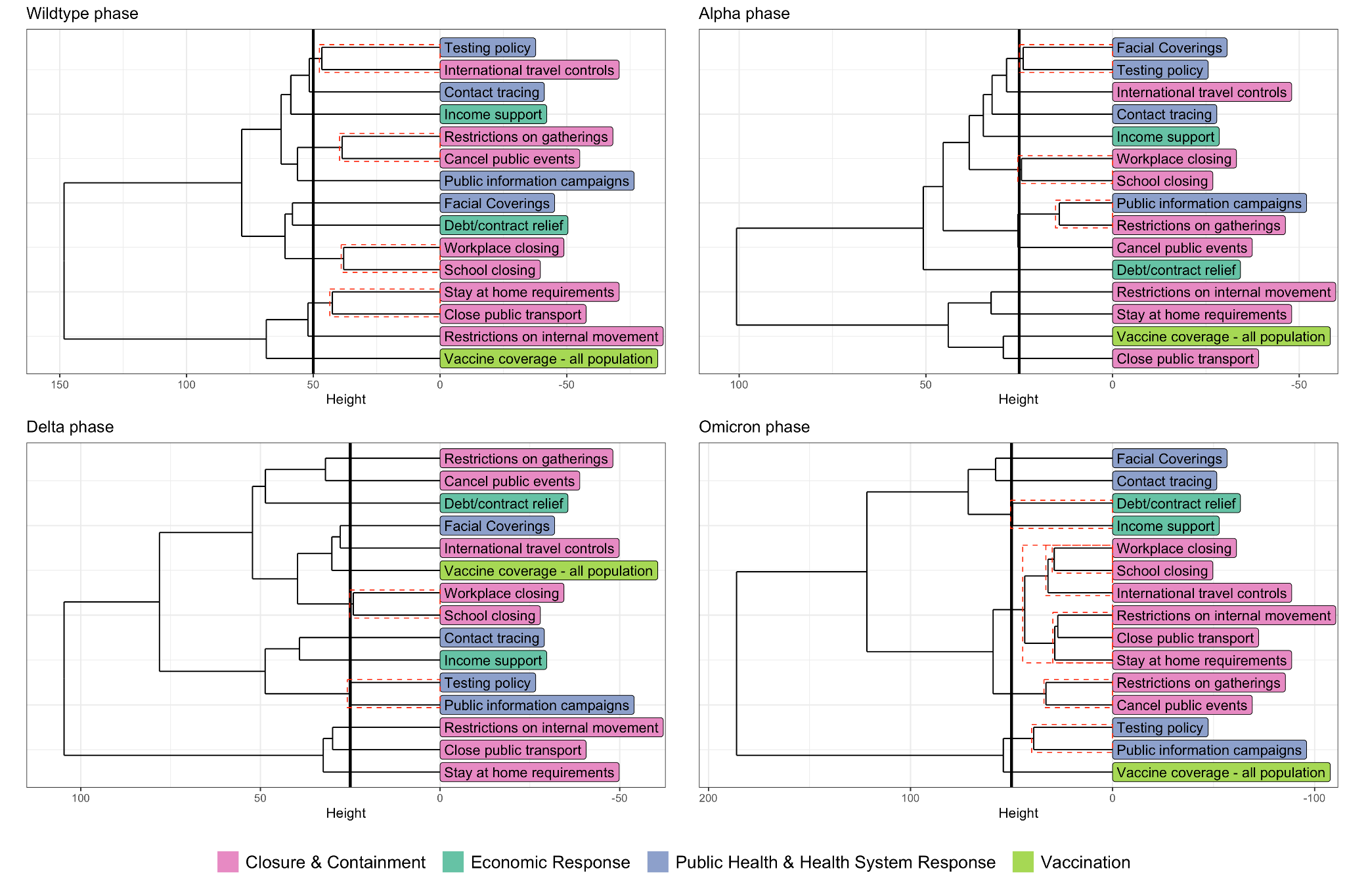


####

### Figure S9: Temporal clusters by variant of concern phases. Dashed red boxes denote statistically significant temporal clusters based on bootstrapping. Detection threshold = 0.4.


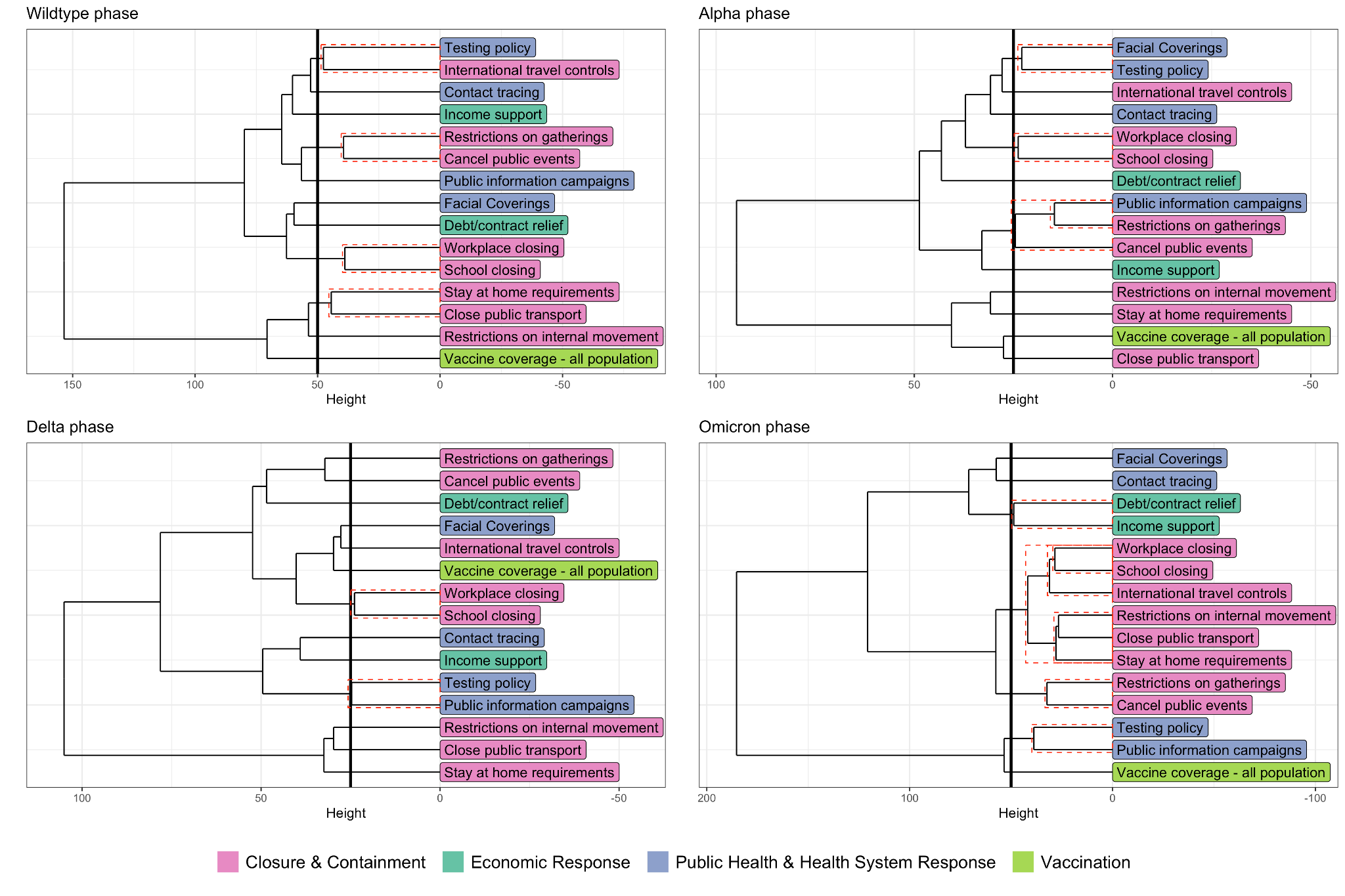


### Figure S10: Temporal clusters by variant of concern phases. Dashed red boxes denote statistically significant temporal clusters based on bootstrapping. Detection threshold = 0.5.


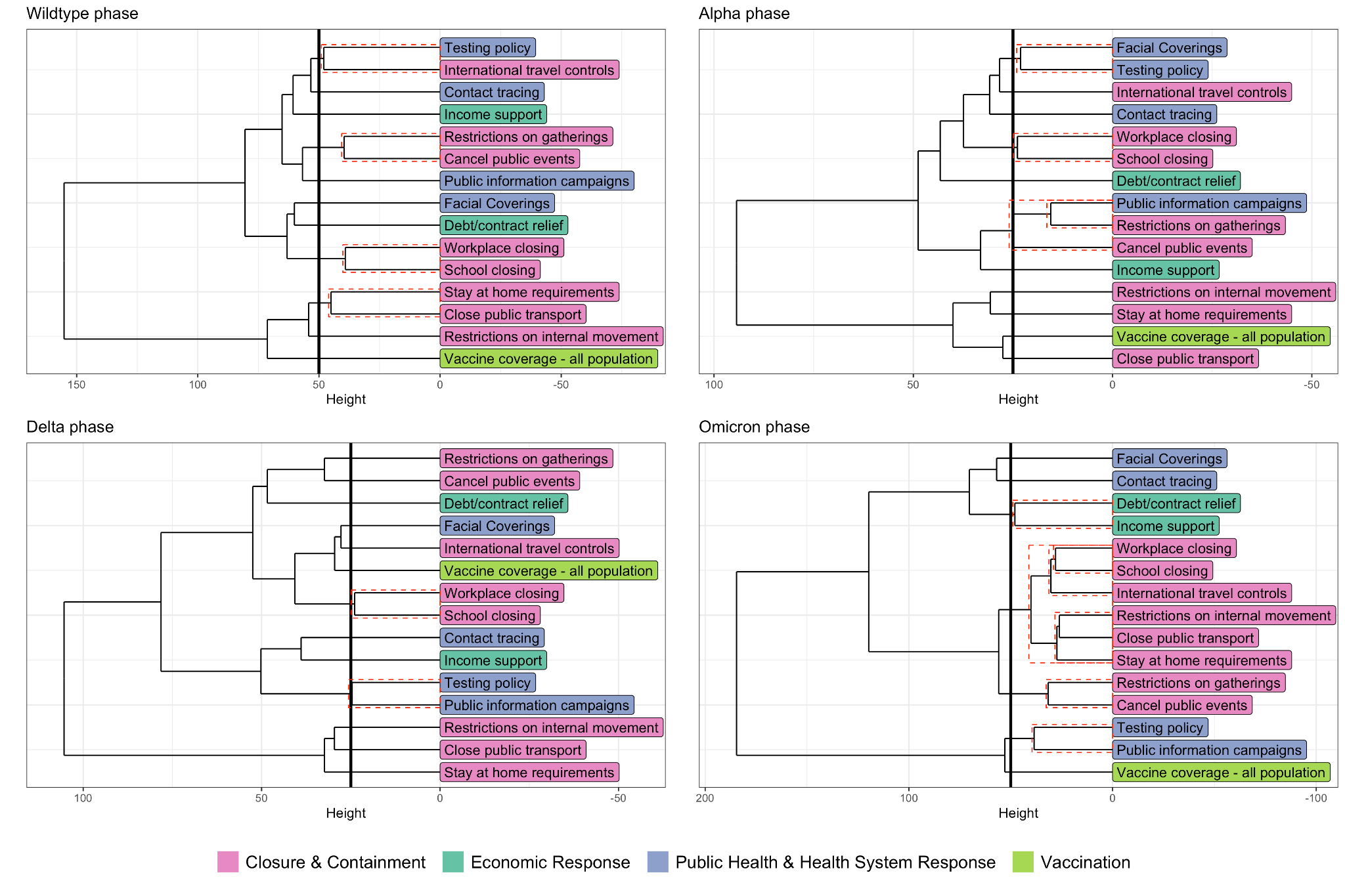


### Figure S11: Effects by variants of concern phases for the association between PHSMs and Rt. Detection threshold = 0.1.


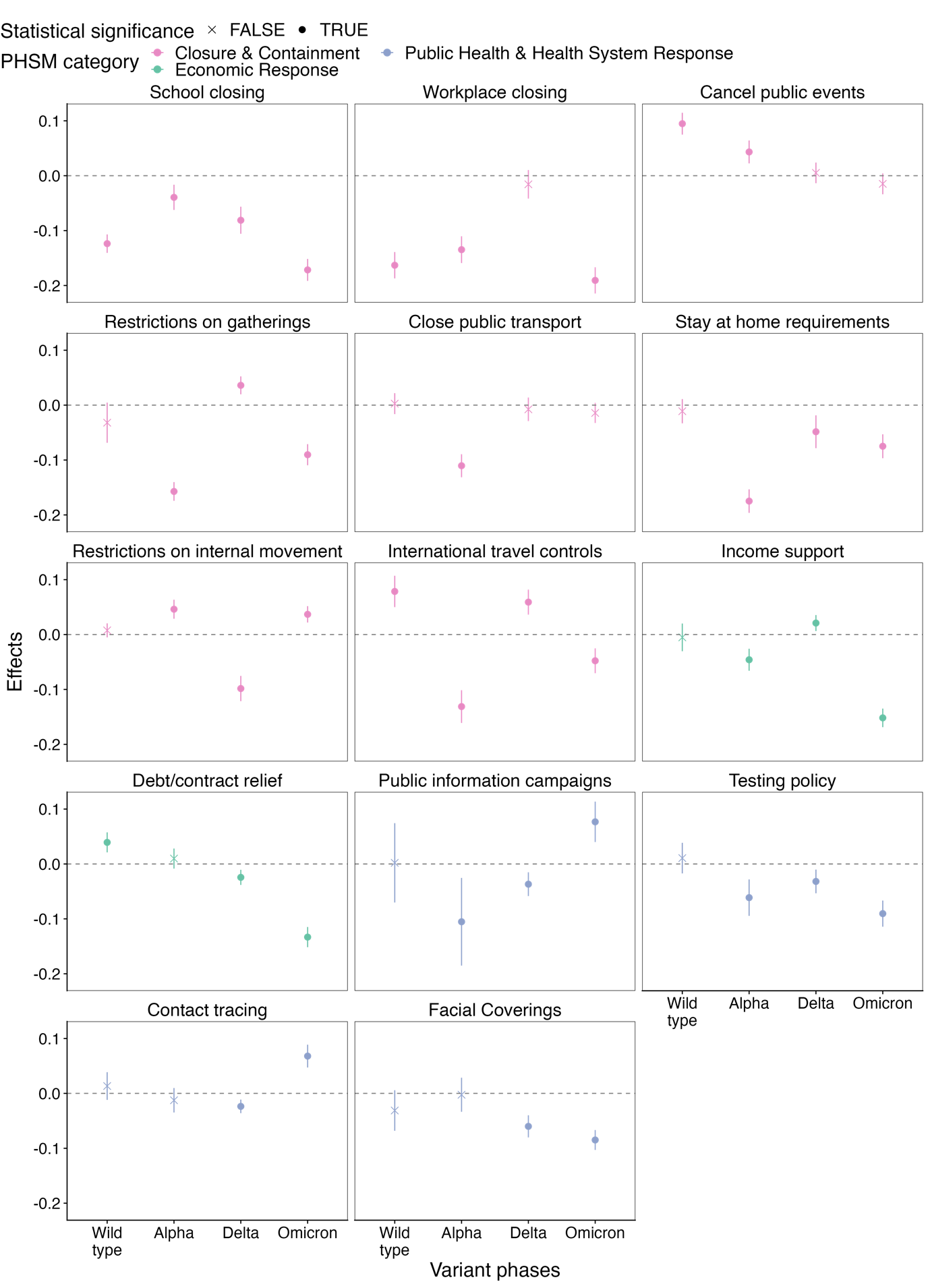


### Figure S12: Effects by variants of concern phases for the association between PHSMs and Rt. Detection threshold = 0.2.


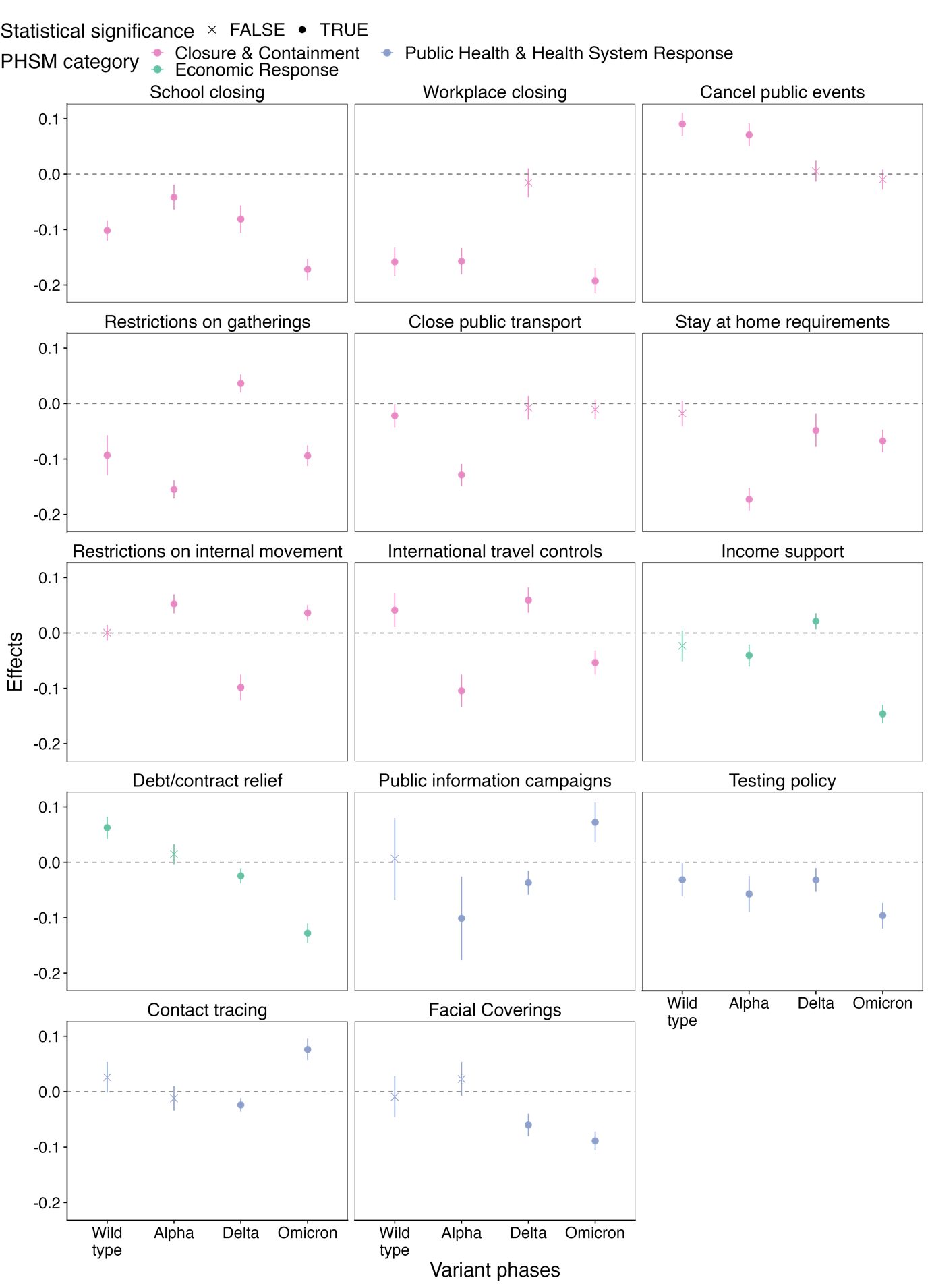


### Figure S13: Effects by variants of concern phases for the association between PHSMs and Rt. Detection threshold = 0.4.


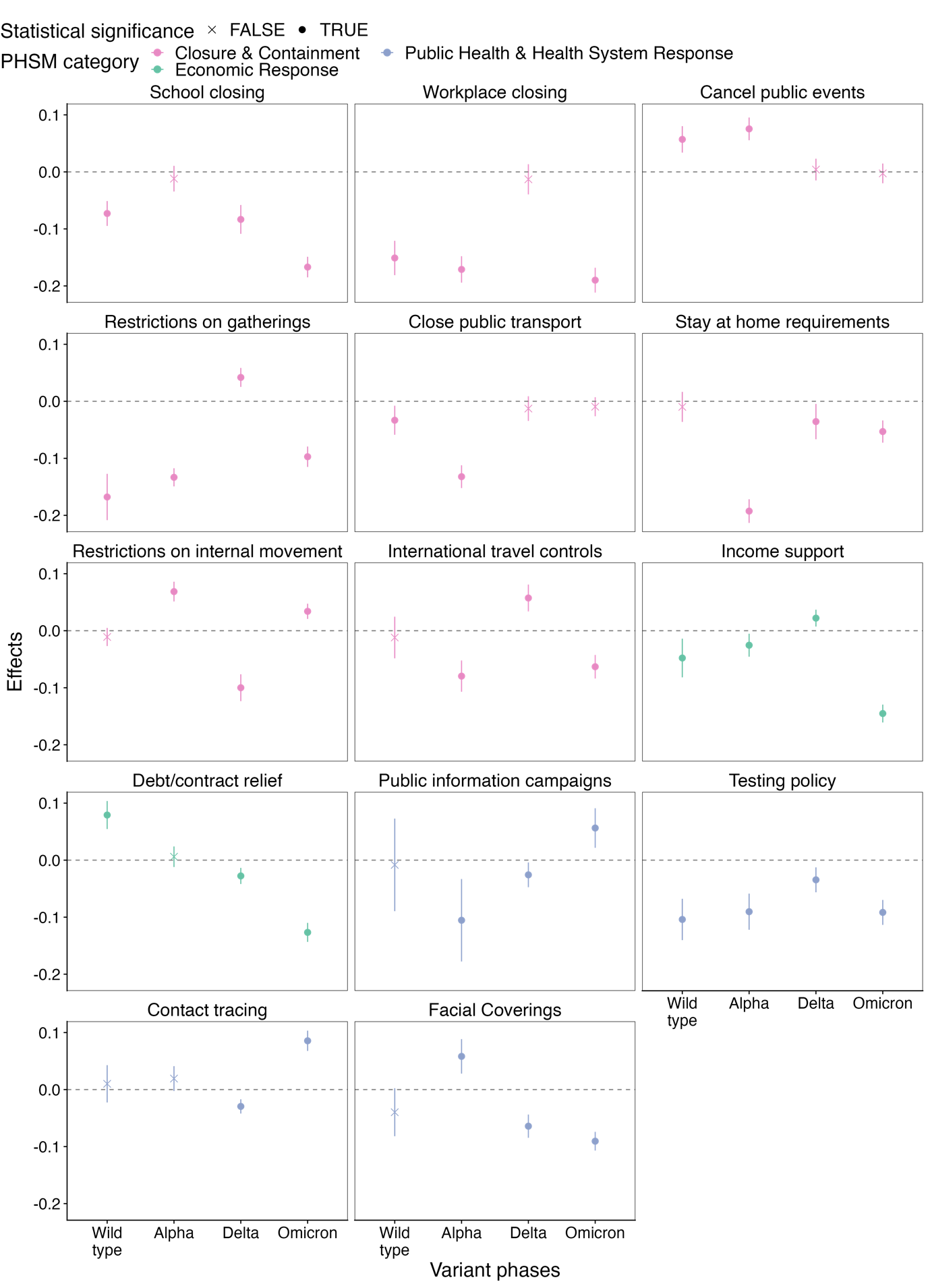


### Figure S14: Effect by variants of concern phases for the association between PHSMs and Rt. Detection threshold = 0.5.


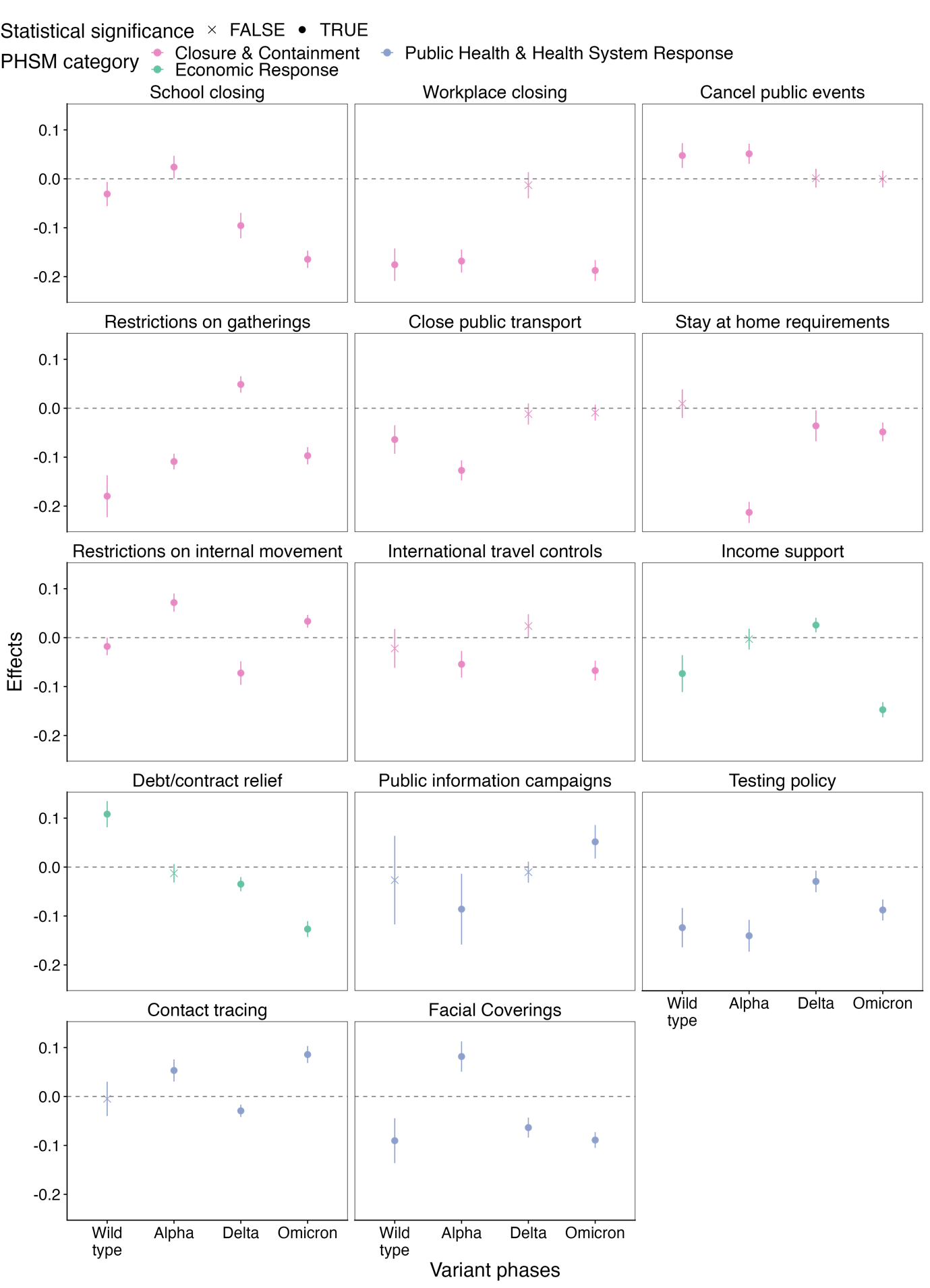


### Figure S15: Effects by variants of concern phases for the association between PHSMs and physical contacts. Detection threshold = 0.1.


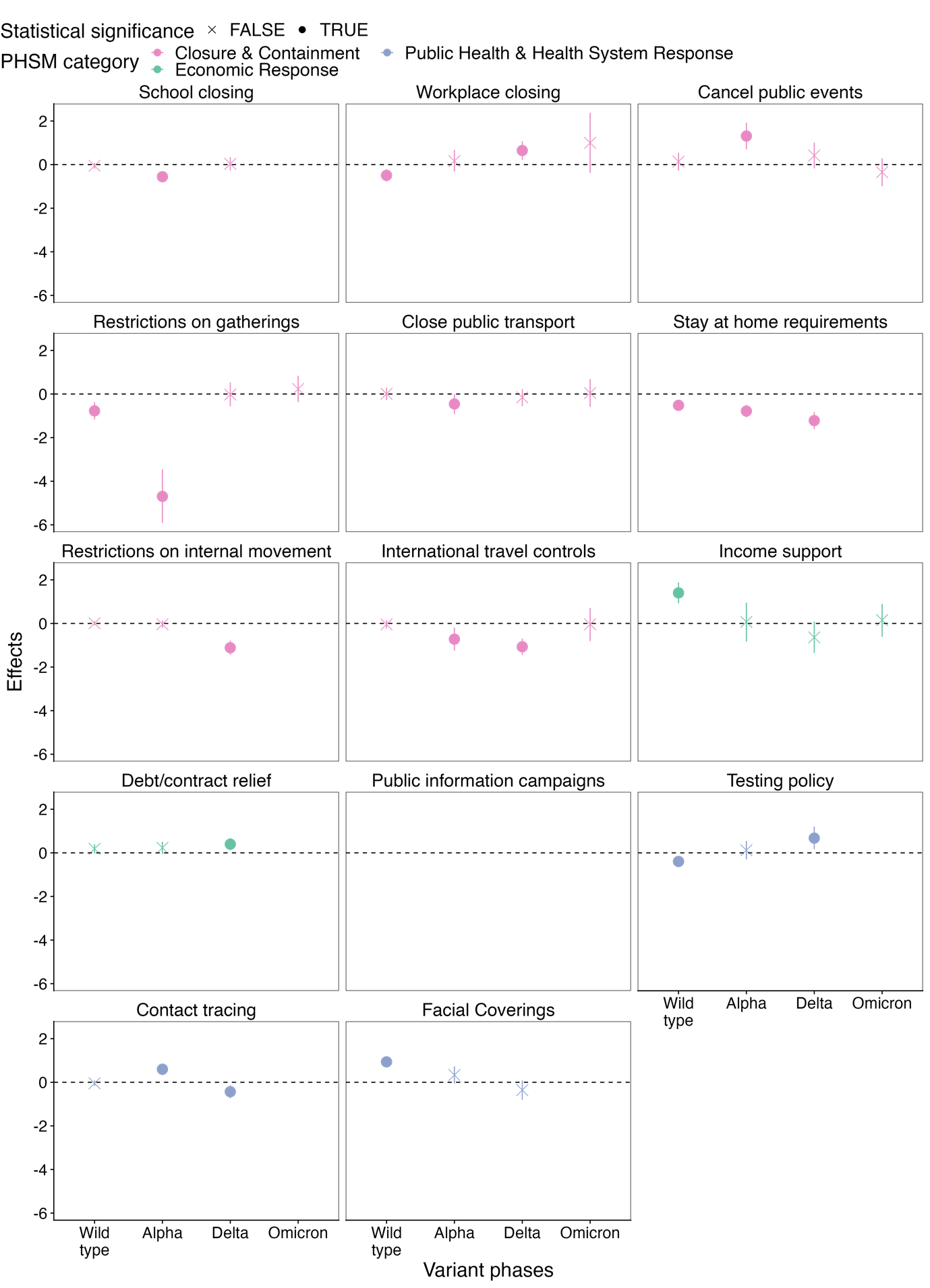


### Figure S16: Effects by variants of concern phases for the association between PHSMs and physical contacts. Detection threshold = 0.2.


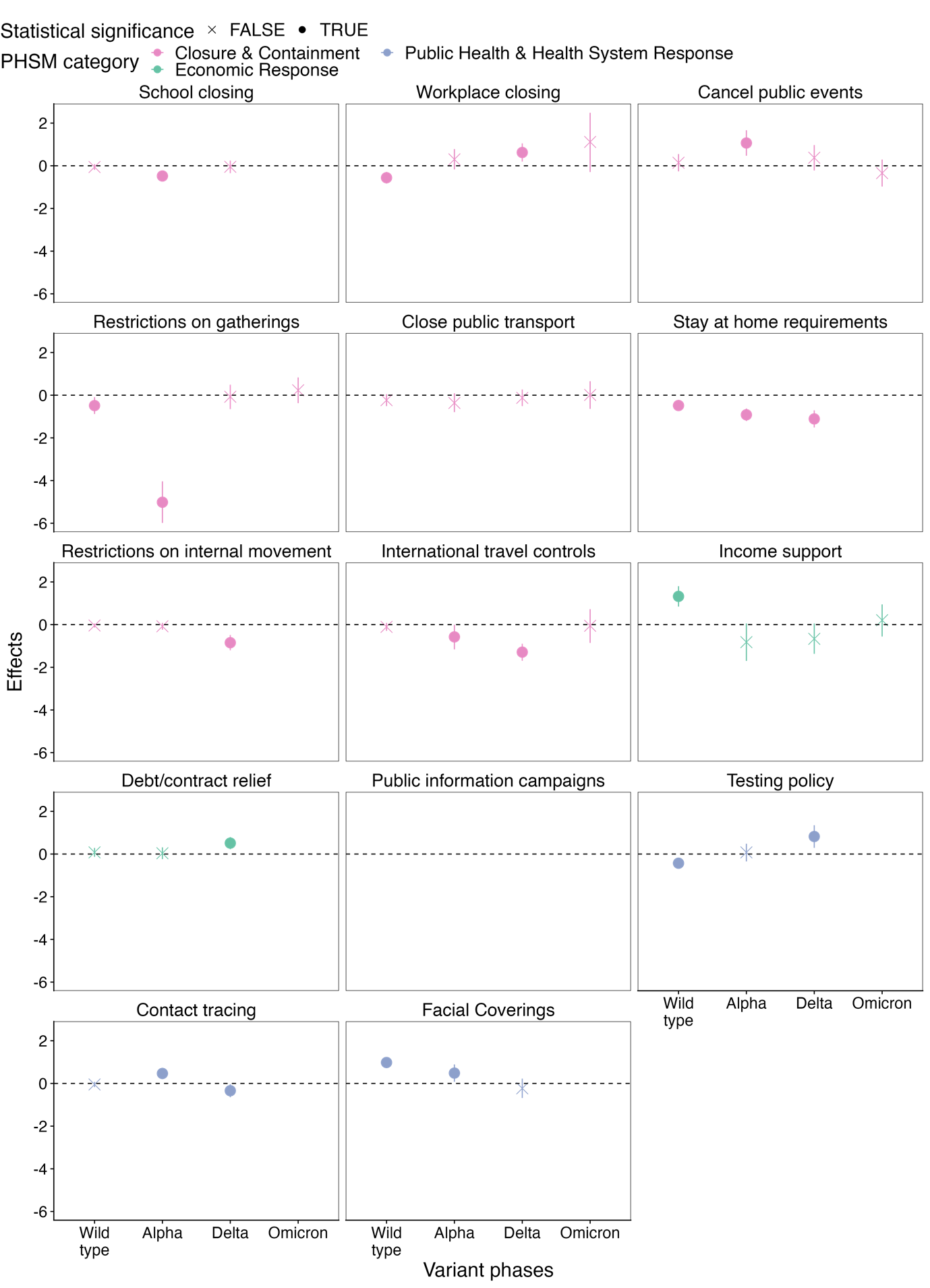


### Figure S17: Effects by variants of concern phases for the association between PHSMs and physical contacts. Detection threshold = 0.4.


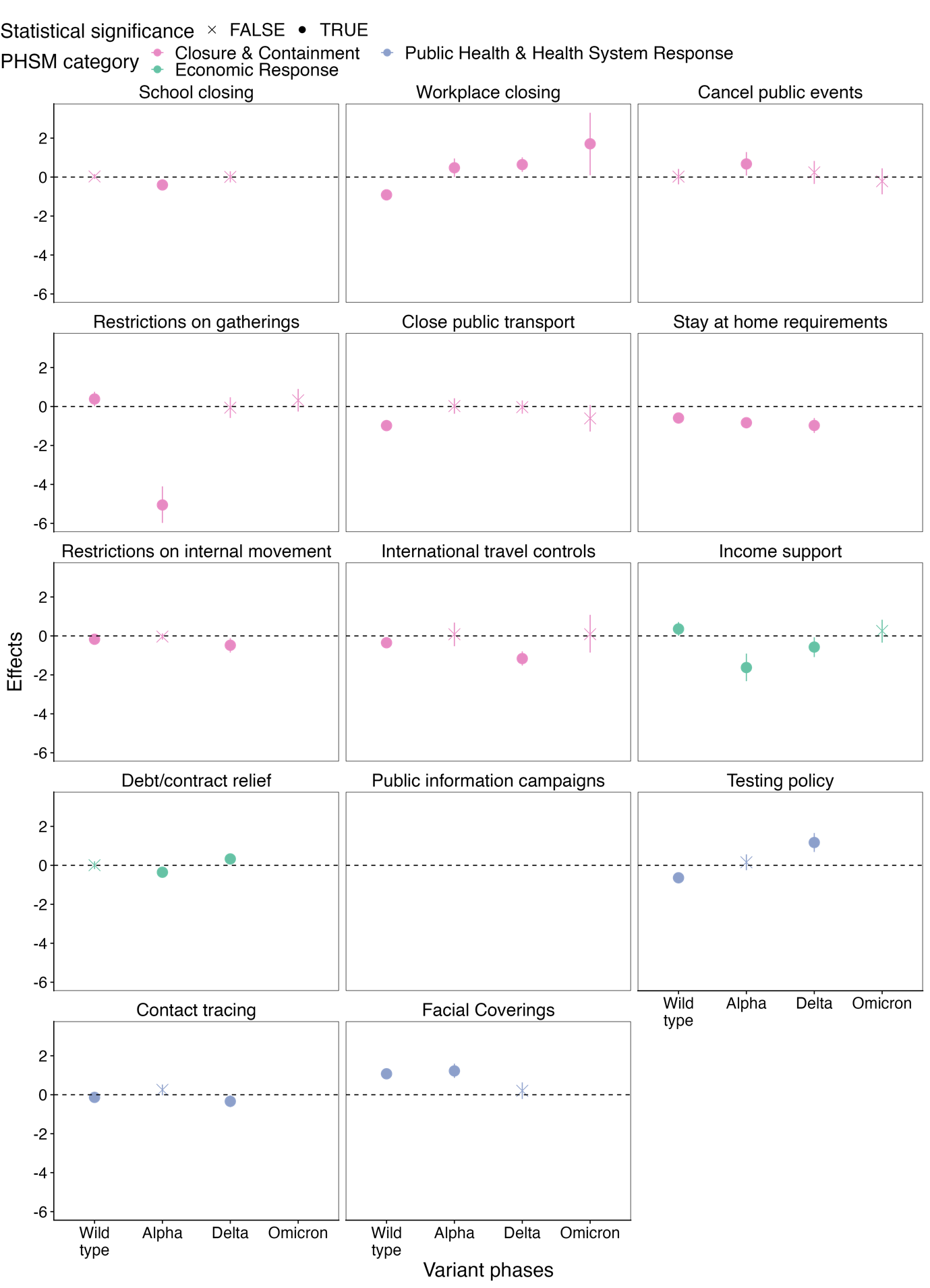


### Figure S18: Effects by variants of concern phases for the association between PHSMs and physical contacts. Detection threshold = 0.5.


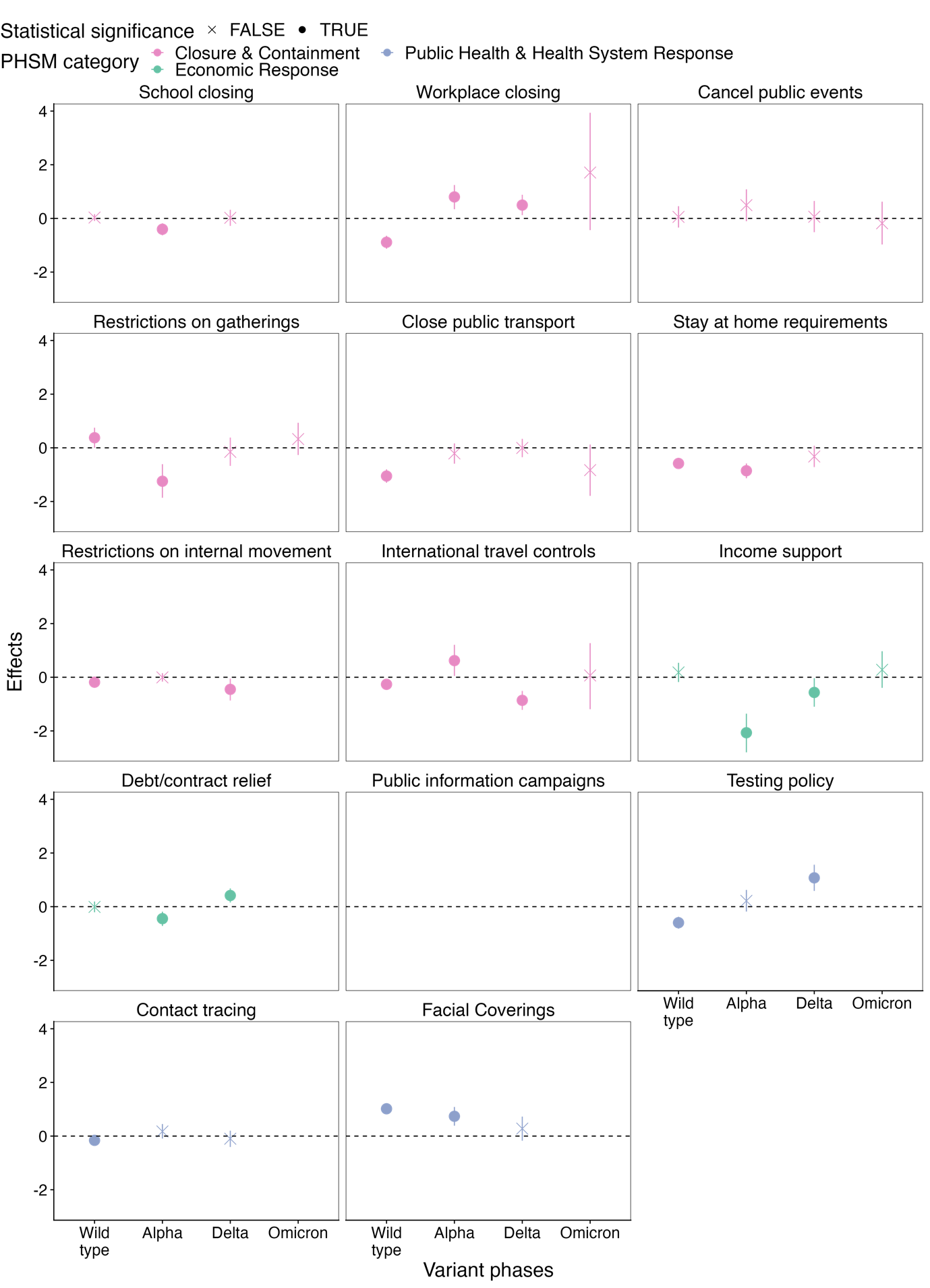

Supplement: Supplementary file 1 — Figure S1: Rt results, panel 1. Countries starting from A to B. Figure S2: Rt results, panel 2. Countries starting from C to G. Figure S3: Rt results, panel 3. Countries starting from G to L. Figure S4: Rt results, panel 4. Countries starting from L to N. Figure S5: Rt results, panel 5. Countries starting from P to S. Figure S6: Rt results, panel 6. Countries starting from S to U. Figure S7: Temporal clusters by variant of concern phases. Dashed red boxes denote statistically significant temporal clusters based on bootstrapping. Detection threshold = 0.1. Figure S8: Temporal clusters by variant of concern phases. Dashed red boxes denote statistically significant temporal clusters based on bootstrapping. Detection threshold = 0.2. Figure S9: Temporal clusters by variant of concern phases. Dashed red boxes denote statistically significant temporal clusters based on bootstrapping. Detection threshold = 0.4. Figure S10: Temporal clusters by variant of concern phases. Dashed red boxes denote statistically significant temporal clusters based on bootstrapping. Detection threshold = 0.5. Figure S11: Effects by variants of concern phases for the association between PHSMs and Rt. Detection threshold = 0.1. Figure S12: Effects by variants of concern phases for the association between PHSMs and Rt. Detection threshold = 0.2. Figure S13: Effects by variants of concern phases for the association between PHSMs and Rt. Detection threshold = 0.4. Figure S14: Effect by variants of concern phases for the association between PHSMs and Rt. Detection threshold = 0.5. Figure S15: Effects by variants of concern phases for the association between PHSMs and physical contacts. Detection threshold = 0.1. Figure S16: Effects by variants of concern phases for the association between PHSMs and physical contacts. Detection threshold = 0.2. Figure S17: Effects by variants of concern phases for the association between PHSMs and physical contacts. Detection threshold = 0.4. Figure S18: Effects by var [file IRV-18-e70036-s001.docx]
